# Giant transposable elements drive genetic diversification in *Aspergillus oryzae*, a domesticated fungus traditionally used in food fermentation

**DOI:** 10.1016/j.isci.2026.117405

**Published:** 2026-09-01

**Authors:** Yuya Hamanaka, Takuya Katayama, Yuta Chiken, Kana Nishiguchi, Katsushi Yamaguchi, Shuji Shigenobu, Jun-ichi Maruyama

**Affiliations:** 1 Department of Biotechnology, The University of Tokyo, 1-1-1 Yayoi, Bunkyo-ku, Tokyo 113-8657, Japan; 2 Collaborative Research Institute for Innovative Microbiology, The University of Tokyo, 1-1-1 Yayoi, Bunkyo-ku, Tokyo 113-8657, Japan; 3 Trans-Omics Facility, National Institute for Basic Biology, Nishigonaka 38, Myodaiji, Okazaki 444-8585, Japan

**Keywords:** *Aspergillus oryzae*, food fermentation, brewing, starship, giant transposable elements, intra-species variation, domestication, artificial selection

## Abstract

*Starships* are giant transposable elements recently reported in fungi that carry cargo genes and drive rapid adaptation. While inter-species variation by *Starships* has been investigated, their role in intra-species variation remains unclear. Here, we investigated *Starships* in *Aspergillus oryzae*, a filamentous fungus differentiated from mycotoxigenic *Aspergillus flavus* and subsequently domesticated for various applications in food fermentation, such as sake, *miso*, and soy sauce production in Japan. Comparative analysis between *A*. *flavus* and *A*. *oryzae* revealed that cargo gene gains/losses during domestication likely contribute to safe and stable food production. Furthermore, cargo gene composition varied even among *A*. *oryzae* strains harboring the same *Starship* classes, suggesting its correlation with specific strain usage. These findings indicate that *Starships* facilitate rapid evolution and diversification under artificial selection by acquiring and rearranging cargo genes. Our study highlights *Starships* as key vehicles driving genetic diversification in the domestication of *A*. *oryzae* for multipurpose industrial applications.

## Highlights

• *Starships* were identified in the domesticated fungus *Aspergillus oryzae*
• Five of the ten identified *Starship* classes are specific to *A*. *oryzae*
• Fermentation-related *Starship* cargo genes are associated with industrial uses
• *Starships* may have contributed to the differentiation and domestication of *A*. *oryzae*

## Graphical abstract

**Figure:**
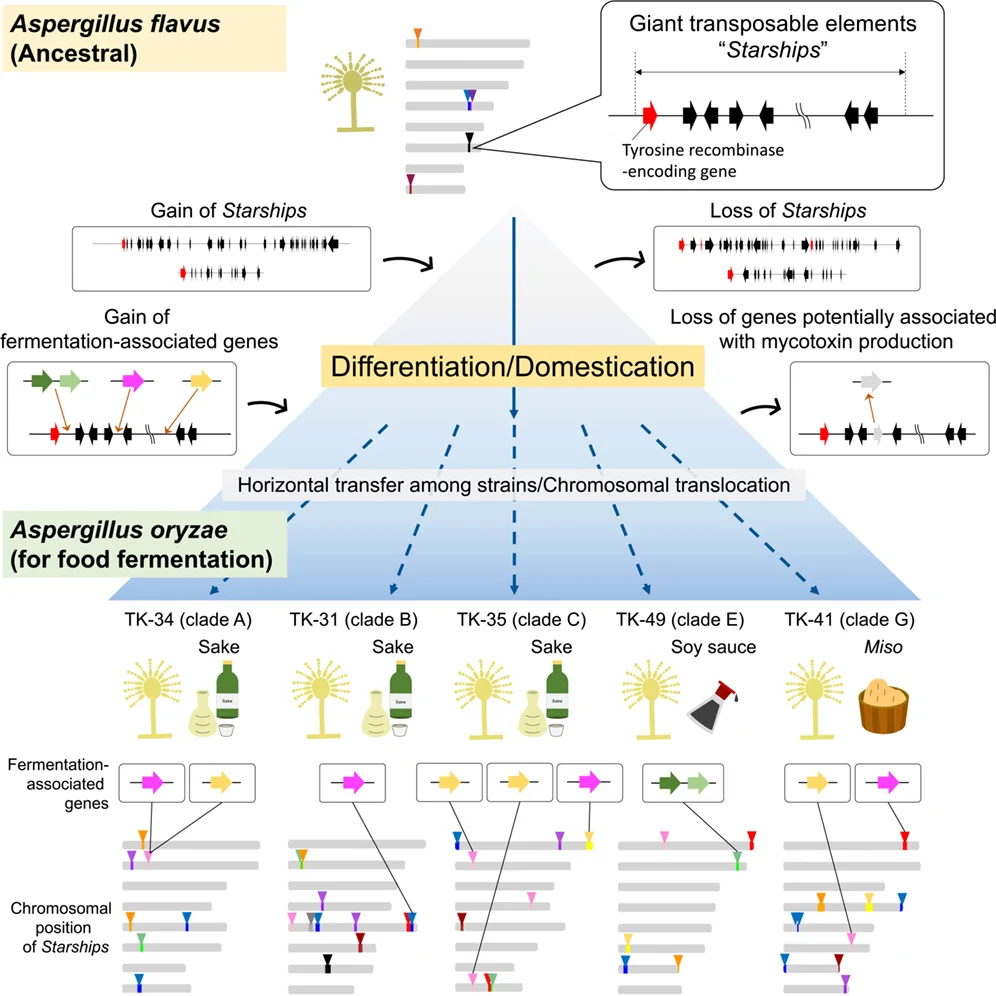

## Teaser

Evolutionary biology; Microbiology

## Introduction

Mobile genetic elements are DNA sequences that move within the genome and are associated with adaptive evolution.^1^^,^^2^ Transposons, one of the mobile genetic elements, are DNA sequences typically ranging from 100 to 10,000 bp that can move to different parts of the genome.^3^^,^^4^ They were first discovered in maize,^5^ and have since been found in almost all organisms, including animals, plants, fungi, bacteria, and archaea.^6^^,^^7^^,^^8^^,^^9^^,^^10^^,^^11^ Transposons can alter fitness by causing loss/gain of gene function or genome rearrangement, such as generating novel regulatory networks or promoting genetic diversity.^12^^,^^13^ Furthermore, there are known cases in which transposons are involved in the domestication process, wherein species possessing traits beneficial to humans are selected.^14^^,^^15^ In bacteria, a distinct type of large mobile genetic element known as integrative and conjugative element (ICE) has been identified.^16^^,^^17^ Unlike typical transposons, ICEs range from 20 to 500 kbp and carry multiple cargo genes that affect the phenotypes of bacterial cells.^16^ Like conventional transposons, ICEs are involved in adaptive traits such as antibiotic resistance through horizontal transfer among species,^18^^,^^19^ playing an important role in generating genomic diversity and promoting adaptive evolution.

In fungi, adaptive evolution is driven by genomic variation via transposons.^20^^,^^21^ Recently, giant transposable elements called *Starships* (ranging from 15 to 700 kbp) were found in the subphylum of filamentous fungi Pezizomycotina.^22^^,^^23^ These elements function as fungal analogs of bacterial ICEs. *Starships* contain tyrosine recombinase (YR) encoding genes at the 5′ terminus of the elements, called “captain genes,” and also contain some genes called “cargo genes.”^22^^,^^23^ It is considered that the YR excises genomic elements from the chromosome and reintegrate them into other chromosome regions.^24^ *Starships* are estimated to be horizontally transferred among inter-genus or inter-species, and contribute to increased host fitness in many filamentous fungal species by conferring traits such as heavy metal and formaldehyde resistance, as well as virulence.^24^^,^^25^^,^^26^^,^^27^^,^^28^^,^^29^^,^^30^ Although *Starships* promote the evolution of inter-species adaptive traits,^25^^,^^27^^,^^29^ their role in the adaptive evolution driven by intra-species strain variation remains largely unexplored.

*Aspergillus oryzae* is a domesticated filamentous fungus that plays an important role in diverse food fermentation processes, including traditional Japanese fermentations such as sake, soy sauce, and *miso* production.^31^ *A*. *oryzae* was differentiated as a lineage from *Aspergillus flavus*, a producer of the carcinogenic mycotoxin aflatoxin, and was subsequently domesticated for food fermentation.^32^^,^^33^ This process was associated with the loss of aflatoxin production and duplication of α-amylase genes.^34^^,^^35^^,^^36^ *A*. *oryzae* includes numerous industrial strains (*Tane Koji* strains, TK strains) developed for specific applications, and these strains are classified into multiple phylogenetic groups, exhibiting high genetic diversity.^37^ However, the genomic basis of strain diversity in *A*. *oryzae* is still poorly understood. Recently, *Starship* elements have been predicted in *A*. *oryzae*^38^^,^^39^; however, their roles have not been well annotated. To investigate the relationship between traits and genomic variation by *Starships*, we generated chromosome-level assemblies of *A*. *oryzae* strains from phylogenetically different groups by long-read sequencing and conducted comparative analysis. Here, we found that *A*. *oryzae* has ten classes of *Starship* elements with diverse distributions among *A*. *oryzae* and *A*. *flavus*. In the same class, some of the *Starship* elements were heterogeneous between *A*. *oryzae* and *A*. *flavus* and among *A*. *oryzae* strains, suggesting that these diversities involve inter- and intra-species variation in *A*. *oryzae*.

## Results

### Genome assembly and comparative analysis of genomic structures among *A*. *oryzae* strains

Chromosome-level assemblies were generated to comparatively analyze the relationship between genomic variation and strain heterogeneity in *A*. *oryzae*. The genomes of 82 *A*. *oryzae* industrial strains, named TK strains, have been previously comprehensively sequenced using short-read sequencing and classified into eight clades.^37^ Strains belonging to clades D and H were not available; therefore, five strains, TK-31, TK-34, TK-35, TK-41, and TK-49, belonging to clades B, A, C, G, and E, respectively, were selected for genome sequencing (Figure S1A). In addition to these strains, the RIB40 strain (belonging to clade F), for which the whole-genome sequence has been determined,^40^ was re-sequenced as a control.

**Figure 1:**
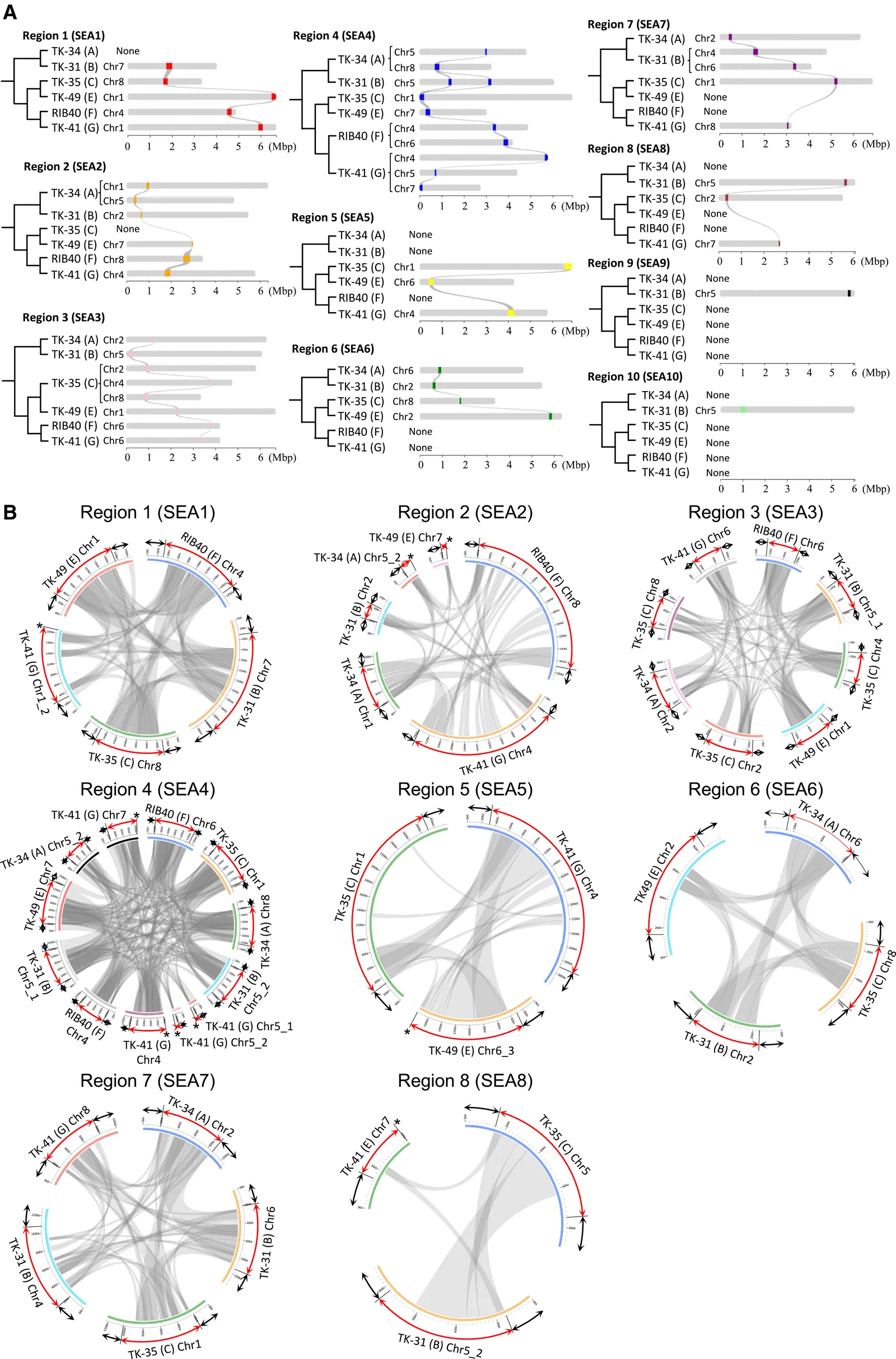
Syntenic genomic regions positioned in different chromosomes of *A*. *oryzae* (A) Chromosome maps of the eight regions in different chromosomes of *A*. *oryzae* strains (regions 1–8) and non-syntenic regions in TK-31 (regions 9 and 10). The colored regions indicate syntenic regions. (B) Circular synteny maps of regions 1–8 and their flanking regions. Red arrows indicate the chromosomal region in Figure 1A, and black arrows indicate its flanking regions. Asterisks represent contig end. Note that there is no synteny in the flanking regions.

**Figure 2:**
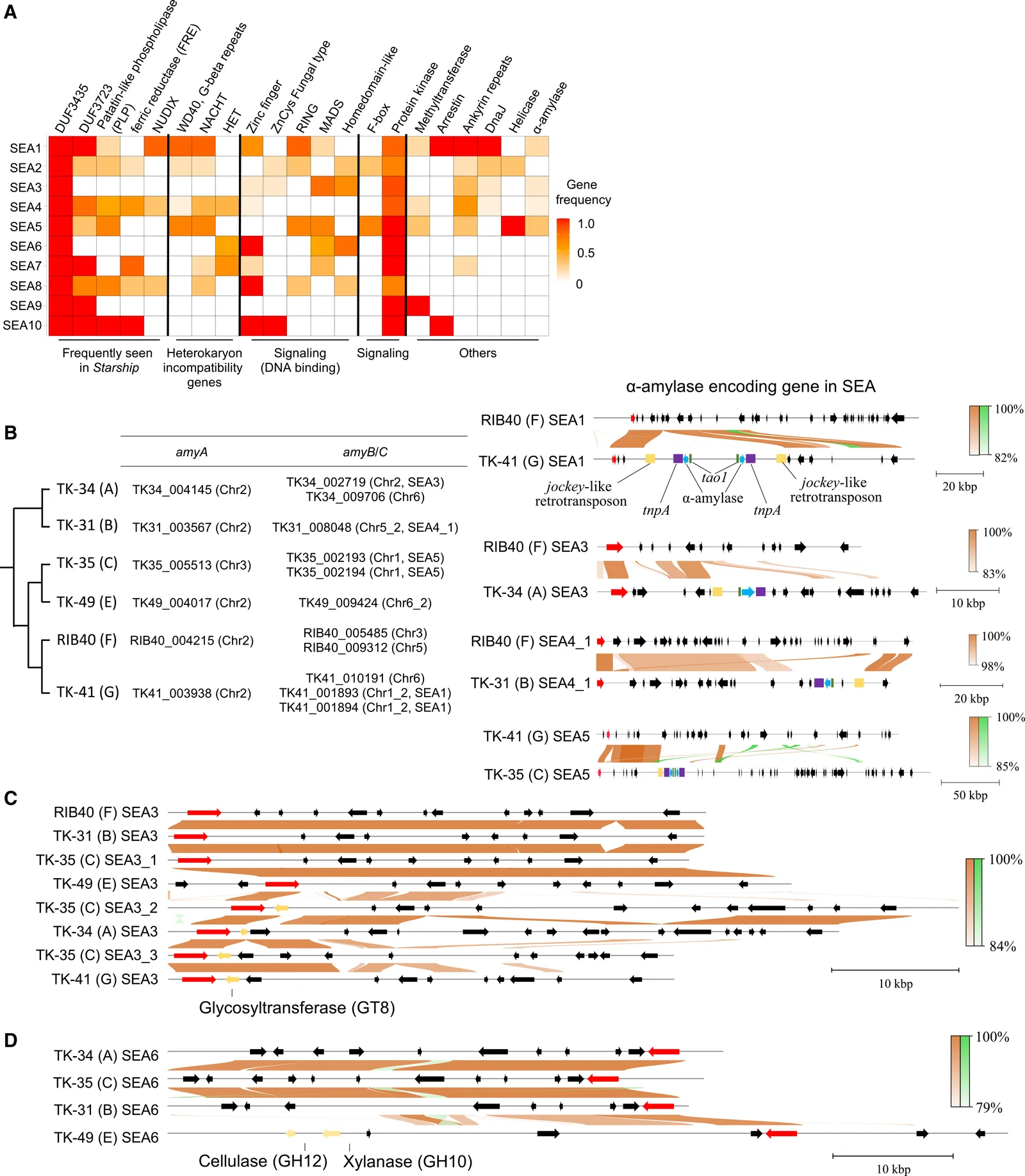
Functional predictions of SEAs (A) Heatmap of gene frequency of SEAs on the basis of domain structures. Gene domain structures were predicted with InterProScan, and the frequencies of genes within each SEA class were quantified. (B) (left) α-amylase genes in *A*. *oryzae* strains. Gene IDs and chromosomal positions of *amyA* and *amyB*/*C* are listed. (right) Synteny maps of strains containing *amyB*/*C* in SEAs. Red and light blue arrows indicate DUF3435-encoding genes and α-amylase genes, respectively. Note that in every SEA region harboring α-amylase, transposons were consistently found in the flanking regions (*jockey*-like retrotransposon, yellow blocks; *tnpA*, purple blocks; *tao1*, green blocks). (C) A synteny map of SEA3. Red and yellow arrows indicate DUF3435-encoding genes and glycosyltransferase 8 (GT8) encoding genes, respectively. GT8 genes were found positioned next to DUF3435-encoding genes. Four of eight SEA3 have GT8 genes. (D) A synteny map of SEA6. Yellow arrow indicates GH12 cellulase or GH10 xylanase encoding genes. GH12 and GH10 genes were present only at the terminal region of the TK-49 strain.

**Figure 3:**
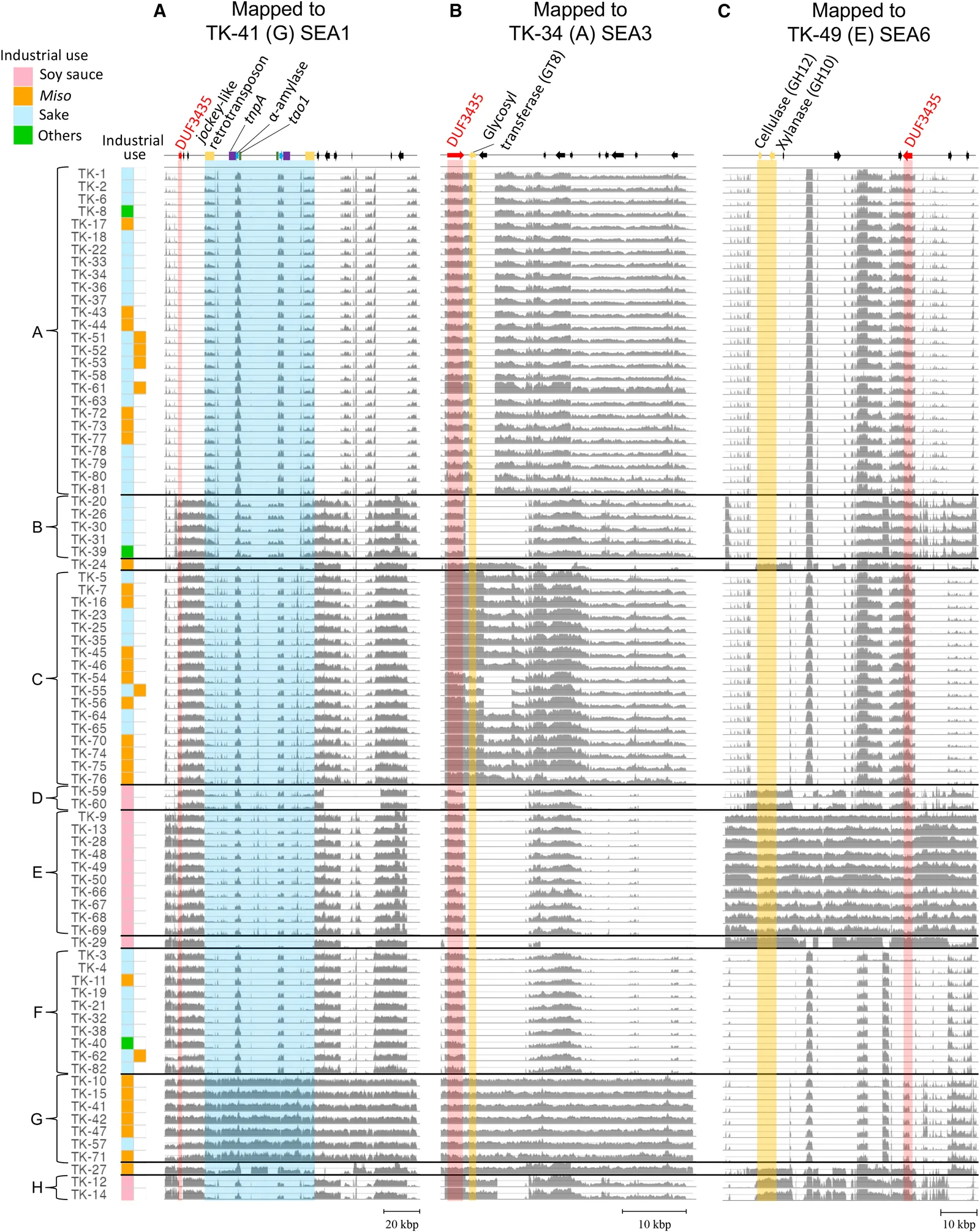
Short-read mapping data for presence or absence of SEAs in *A*. *oryzae* (A) Short-read sequences (BioProject no. PRJDB7763)^37^ mapped to sequences of TK-41 SEA1. Industrial uses of each strain based on Watarai et al.^37^ are indicated. Red highlights/arrows represent DUF3435-encoding gene. The light blue arrow indicates α-amylase gene, and light blue highlight represents α-amylase gene and its flanking regions. TK-41 SEA1 contains *jockey*-like retrotransposon (yellow blocks), *tnpA* transposon (purple blocks), and *tao1* transposon (green blocks). Note that mapped reads were detected in the regions corresponding to the α-amylase gene and transposons; however, this mapping is likely attributable to the presence of multiple similar sequences within the genome. In contrast, reads mapped across the entire α-amylase gene together with its flanking regions were present only in clade G, which includes TK-41. (B) Short-read sequences mapped to sequences of TK-34 SEA3. Red highlights/arrows represent DUF3435-encoding gene. Yellow highlights/arrows represent GT8 genes. Note that in clades A, C, G, and H, reads were mapped to the regions containing the GT8 gene. (C) Short-read sequences mapped to sequences of TK-49 SEA6. Red highlights/arrows represent DUF3435-encoding gene. Yellow highlights/arrows represent GH10 and GH12 genes. Note that in clades D, E, and H, reads were mapped to the regions containing the GH10 and GH12 genes.

**Figure 4:**
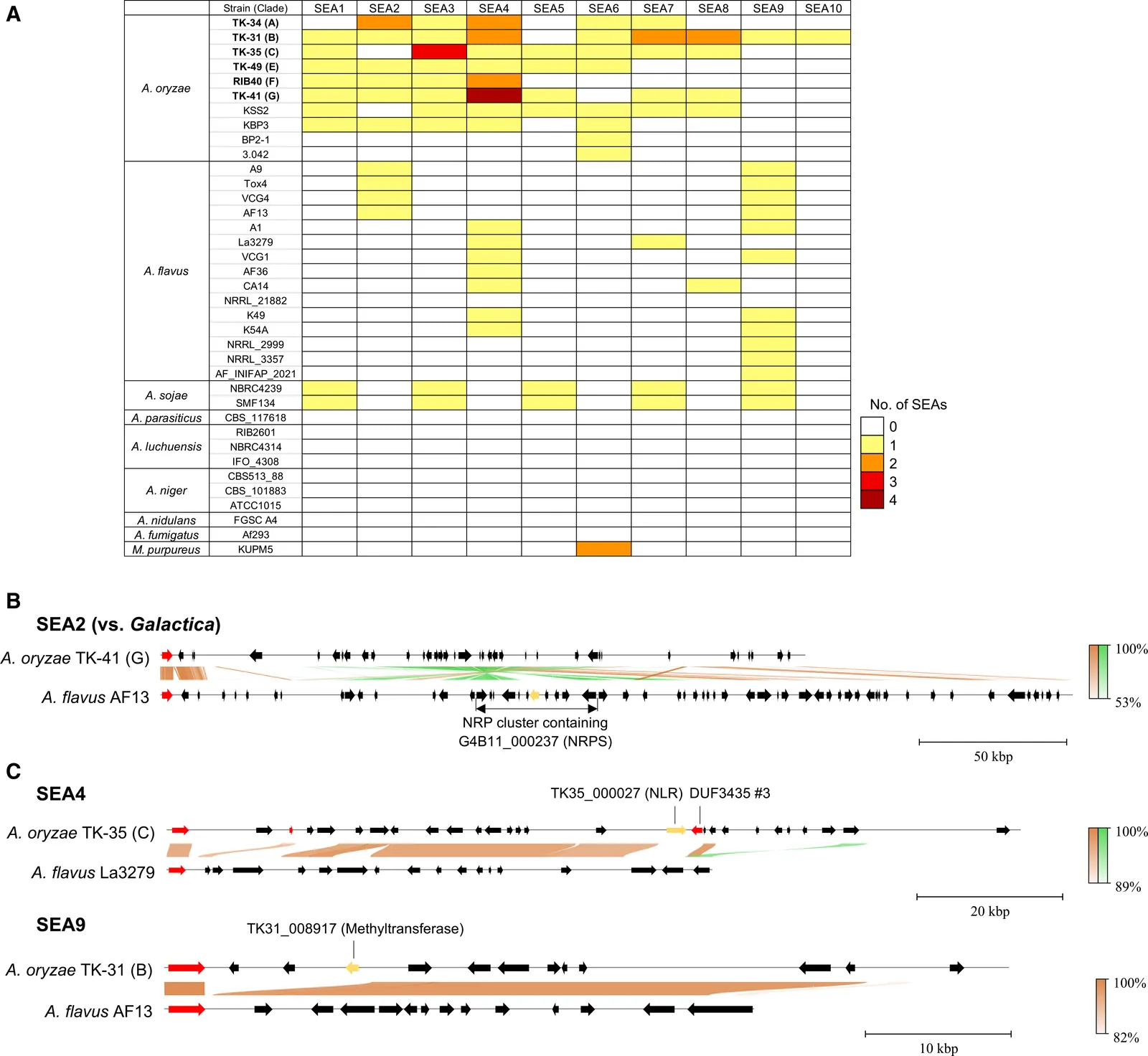
Distribution of 10 classes of SEAs in filamentous fungi (A) Heatmap of number of SEAs in filamentous fungal strains. Based on BLASTn analysis, genomic regions with hits above 30% in “query cover” and with DUF3435-encoding genes were considered to indicate the presence of SEAs. (B) Synteny analysis of SEA2 containing *A*. *flavus*-specific genes. SEA2 in TK-41 and *Galactica* in *A*. *flavus* AF13 (Chr1, 607,240–916,837 bp) were compared. Note that the *NRPS* gene and flanking regions of *A*. *flavus* were absent in *A*. *oryzae*. (C) Synteny analysis of SEA4 and SEA9 containing *A*. *oryzae*-specific genes. SEA4 in TK-35 and *A*. *flavus* La3279 (Chr4, 3,364,977–3,447,713 bp), and SEA9 in TK-31 and *A*. *flavus* AF13 (Chr6, 3,335,948–3,376,442 bp) were compared, respectively. Note that the NLR gene and methyltransferase gene were present in *A*. *oryzae*, but absent in *A*. *flavus*.

**Figure 5:**
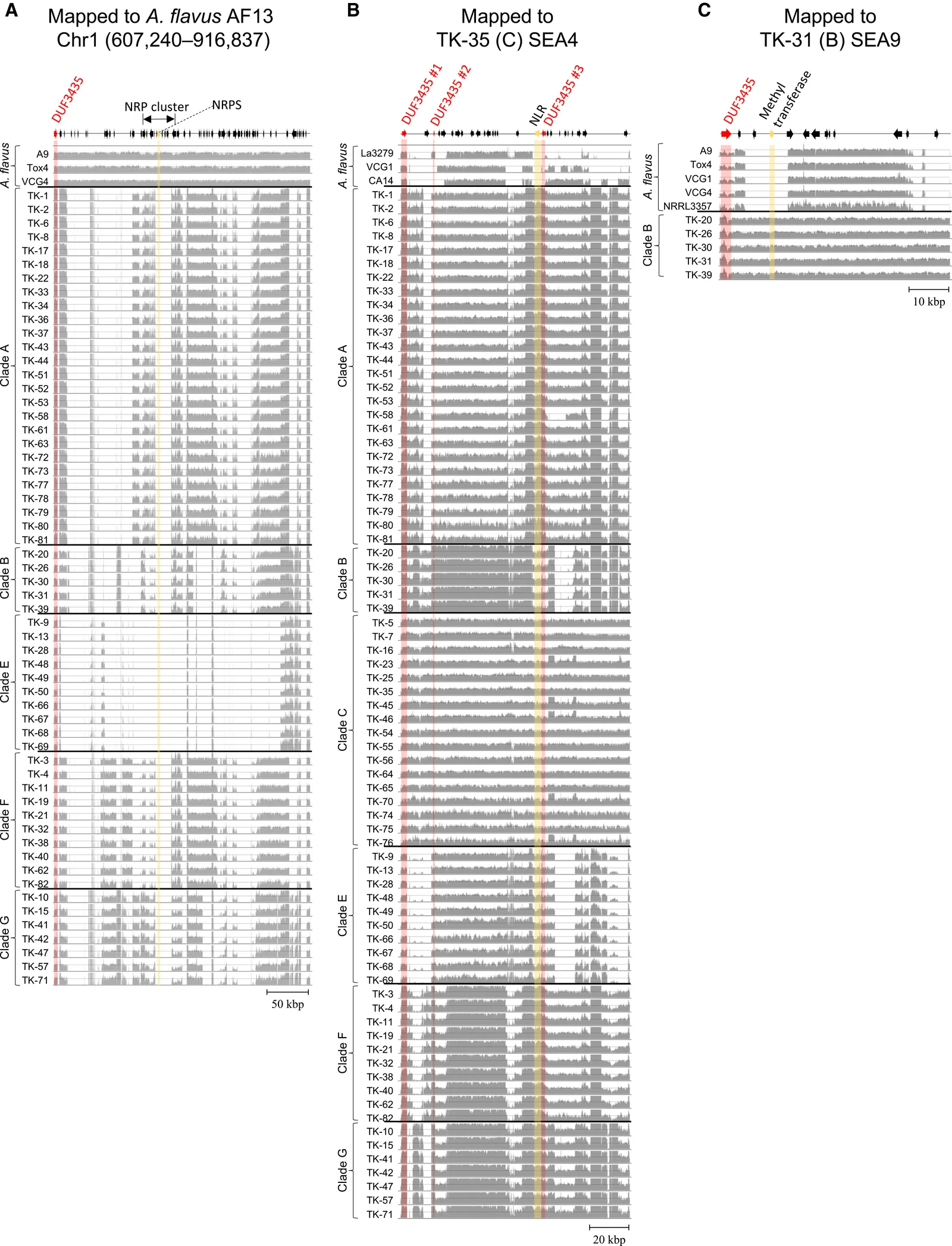
Short-read mapping data for presence or absence of SEAs in *A*. *oryzae* and *A*. *flavus* (A) Short-read sequences (BioProject no. PRJDB7763)^37^ mapped to sequences of *A*. *flavus* AF13 chromosome 1 (607,240–916,837). Red highlights/arrows represent DUF3435-encoding gene. The yellow arrow/highlight indicates an *NRPS* gene. Note that reads were not mapped to *NRPS* gene and its flanking regions in *A*. *oryzae*. (B) Short-read sequences mapped to sequences of TK-35_SEA4. Red highlights/arrows represent DUF3435-encoding gene. Yellow highlights/arrows represent NLR genes. Note that reads were not mapped to regions containing NLR genes in *A*. *flavus*. (C) Short-read sequences mapped to sequences of TK-31_SEA9. Red highlights/arrows represent DUF3435-encoding gene. Yellow highlights/arrows represent methyltransferase genes. Note that reads were not mapped to regions containing methyltransferase genes in *A*. *flavus*.

**Figure 6:**
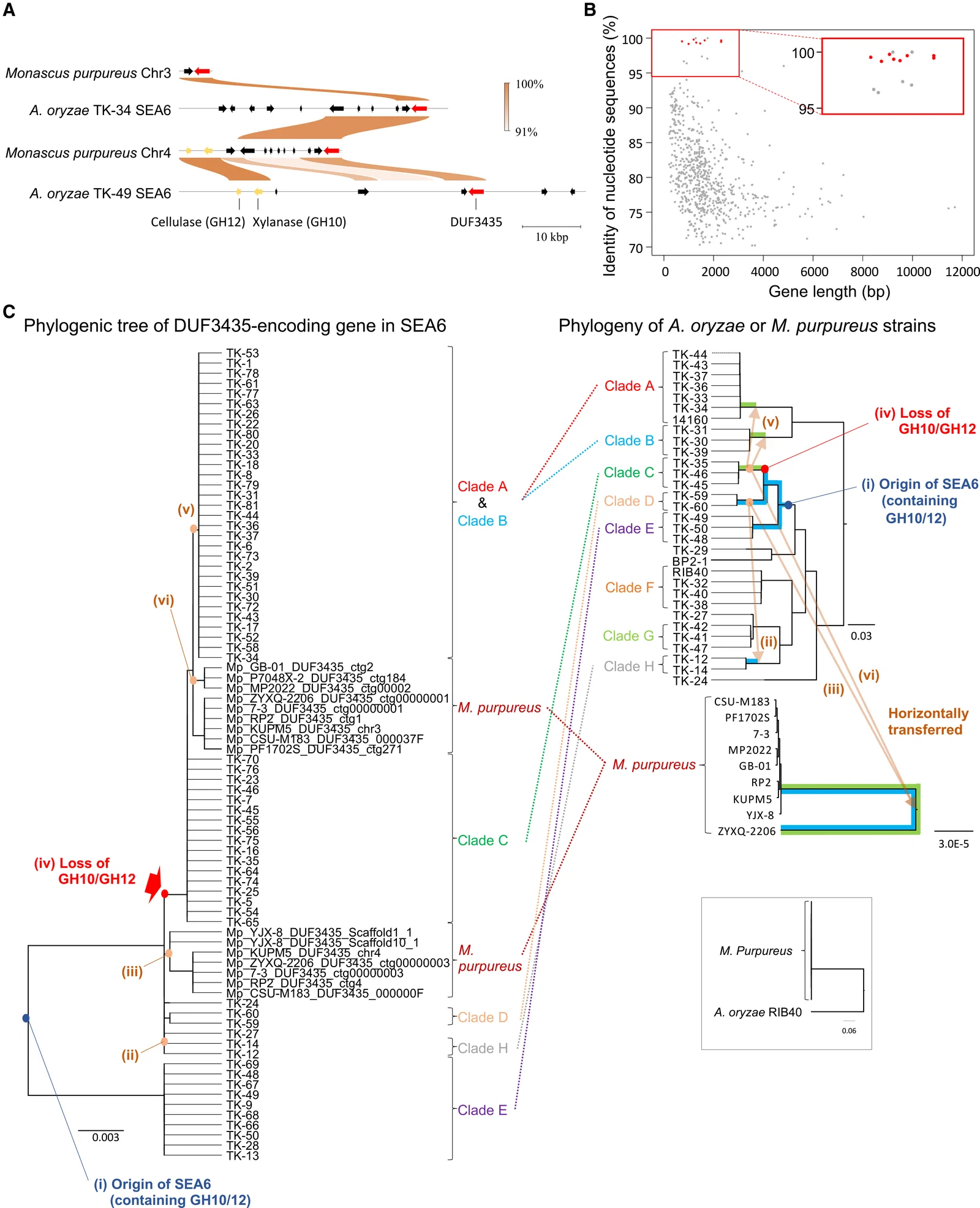
Putative horizontal transfer between *A*. *oryzae* and *M*. *purpureus* (A) Synteny map of SEA6 in *A*. *oryzae* and *M*. *purpureus*. Genome sequences of SEA6 in *A*. *oryzae* (TK-34 and TK-49) and chromosomes 3 and 4 in *M*. *purpureus* KUPM5 strain (acc. no. ASM2599979v1) were compared. Red arrows indicate DUF3435-encoding genes, and yellow arrows represent GH10 or GH12 genes. Note that GH10 and GH12 genes were only present within SEA6 of *A*. *oryzae* TK-49 and Chr3 of *M*. *purpureus*. (B) Gene similarity between *M*. *purpureus* KUPM5 and *A*. *oryzae* TK-49. The open reading frame sequences of the genes in TK-49 were subjected to a BLASTn search against *M*. *purpureus*, and the identity was calculated. Each plot represents each hit against KUPM5. Red plot indicates genes in SEA6 of TK-49. (C) Evolutionary model of transposition of SEA6 in *A*. *oryzae*. The most plausible evolutionarily model, minimizing the number of gene gain and loss events, was estimated. Horizontal transfer was considered only between clades, excluding transfer among strains. (Left) Phylogenic tree of DUF3435-encoding gene in SEA6. Following the inferred evolutionary order, the origin of SEA6 (blue), the horizontal transfer of SEA6 (brown), and the loss of GH10 and GH12 genes (red) are indicated using numbers. (Right) Phylogenetic tree of *A*. *oryzae* strains modified from Figure S1. Phylogenetic tree of *M*. *purpureus* strains and *A*. *oryzae* RIB40 were constructed. The blue/green lines indicate that SEA6 was retained in *A*. *oryzae* strains belonging to clades C, D, and E, and that the element was horizontally transferred to *M*. *purpureus*. The blue line represents SEA6 containing GH10 and GH12 genes, whereas the green line represents SEA6 without GH10 and GH12 genes.

**Figure 7:**
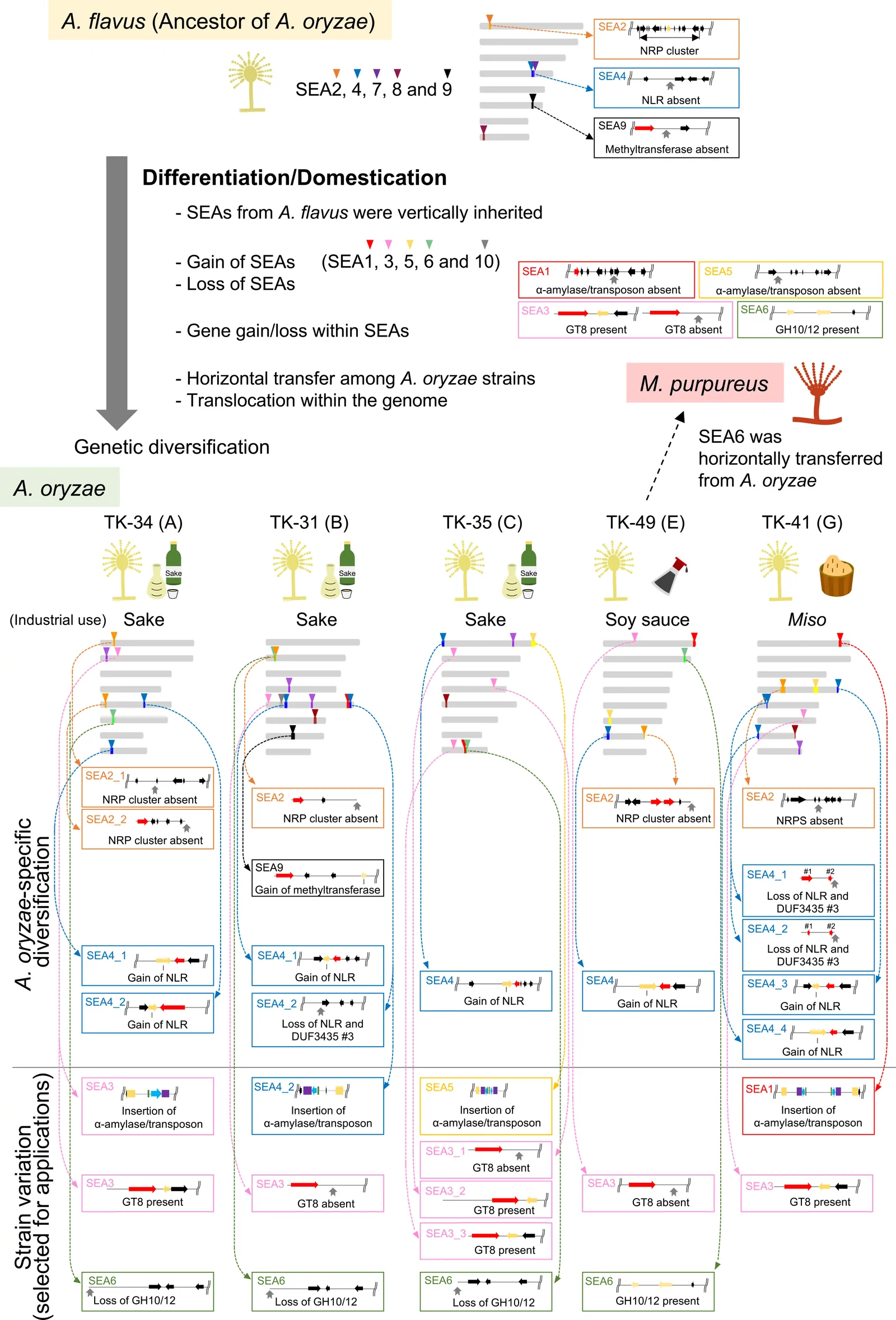
Conceptual model of SEAs spreading among *A*. *oryzae* strains The ancestral *A*. *flavus* lineage carried several SEAs, including SEA2, SEA4, SEA7, SEA8, and SEA9. Their chromosomal positions in the ancestral *A*. *flavus* genome were inferred from those in representative *A*. *flavus* strains. During differentiation from *A*. *flavus* to *A*. *oryzae* and its subsequent domestication, some SEAs were vertically inherited. Additional SEAs, including SEA1, SEA3, SEA5, SEA6, and SEA10, were acquired in the *A*. *oryzae*, whereas some SEAs appear to have been lost. Likewise, some cargo genes such as the NRP cluster genes, were lost; whereas, others, including NLR, methyltransferase, α-amylase, GT8, and GH10/12 genes, were acquired. Furthermore, genomic rearrangements also occurred through horizontal transfer among *A*. *oryzae* strains and translocation within individual genomes. These processes generated variation in SEA and cargo-gene composition among strains, some of which may subsequently have been selected during their use for specific industrial applications. Representative strains used for sake, soy sauce, and *miso* production, therefore, exhibit characteristic SEA compositions. SEA6 was also horizontally transferred from *A*. *oryzae* to *M*. *purpureus*.

The *de novo* assembly of the six *A*. *oryzae* strains showed 37.3–39.2 Mbp in 8–13 contigs (Figure S1B; Table S1). In addition, 98.0%–98.5% of the BUSCO values indicated high completeness. In particular, the genome sequences of TK strains were more contiguous than that obtained with sequencing using short-read technology.^37^ The chromosome of assemblies in TK strains was defined based on whole chromosomal alignment between the RIB40 strain and five TK strains (Figure S1C). Most contigs from the TK strain aligned uniquely to contigs of the RIB40 strain (Figure 1, Figure 2, Figure 3, Figure 4, Figure 5, Figure 6, Figure 7S1C), suggesting that chromosomal structures were nearly conserved among the *A*. *oryzae* strains. However, some contigs separately aligned to multiple contigs of the RIB40 strain, and thus, chromosomal terminal regions (0.3–1.0 Mbp) were swapped between the TK strains and RIB40 (Figure S1C: black arrowheads). Comparison of the same chromosomal regions between the genomic sequences of *A*. *oryzae* strains using synteny analysis (for example, Chr. 4 in RIB40 and TK-35; Figure S2A) revealed that eight chromosomal regions of approximately 10–290 kbp were positioned on different chromosomes between the strains (Figure 1A; regions 1–8). Despite being located at different positions on the chromosome, these regions of the same class exhibited high sequence similarity among *A*. *oryzae* strains, although some strains had distinct sequences in part (Figure 1B). In some of the chromosomal regions, two or three were present in a single strain, while some strains did not have such chromosomal regions (Figure 1A). Notably, the DUF3435 domain-encoding gene was positioned at the end of these chromosomal regions (Figure S2A). Recently, the DUF3435-encoding gene was reported to commonly exist in *Starships*.^22^^,^^23^ Therefore, we designated the chromosomal regions containing DUF3435-encoding genes as *Starship* Element in *A*. *oryzae* (SEA1–8).

### Classification of giant transposable *Starship* elements in *A*. *oryzae*

In the above investigation, we focused on regions existing at different chromosomal positions among the strains, but this may potentially ignore chromosomal regions containing DUF3435-encoding genes. Hence, we comprehensively explored genes containing the DUF3435 domain (PF11917 or IPR021842) and identified 66 DUF3435-encoding genes in the *A*. *oryzae* genomes of the six strains. Phylogenetic analysis classified the 66 DUF3435-encoding genes into 12 clades (Figure S2B). Genes belonging to 10 clades of 12 clades were included in SEA1–8 classes, in which SEA4 class contained additional two clades of DUF3435-encoding genes (SEA4 #2 and #3 in addition to SEA4 #1). Two chromosomal regions containing remaining clades of DUF3435-encoding genes were found specifically in the TK-31 strain; these two regions were designated as SEA9 and SEA10 classes (Figure 1A). Eventually, 48 SEAs (across SEA1 to SEA10 classes) were identified among the six *A*. *oryzae* strains (Figure S3). The SEAs ranged from 6.3 kbp to 288.8 kbp and had an average GC content of 45.0% (Table S2), while the regions classified into the same SEAs were largely homologous despite some deletion or insertion of genes (Figure S3). Terminal inverted repeat (TIR) and direct repeat (DR) sequences are often found at both ends of *Starship* elements and are involved in facilitating the transposition process.^23^ Hence, TIR and DR sequences were predicted in the identified SEAs, and their sequences were found to differ depending on the SEA class (Figure S4), supporting the classification of the SEAs.

Previous studies have revealed that *Starship* elements contain the DUF3435-encoding genes at their terminal parts, which are called “captain,” as they mediate the translocation of the *Starship* element containing cargo genes.^22^^,^^23^ Consistent with these findings, most of the identified SEAs contained the DUF3435-encoding genes at their terminal parts (Figure S3). Although some strains possessed two or three DUF3435-encoding genes in SEA4, the DUF3435-encoding genes belonging to the SEA4 #1 clade (Figure S2B) were defined as captain genes based on their positions in the regions (Figure S3). *Starship* elements were classified based on the DUF3435-encoding gene phylogeny.^38^ BLASTn search of DUF3435-encoding genes against the *starbase*, which is a database of *Starship* elements,^41^ revealed that SEA1, SEA3, SEA6, SEA7, and SEA8 belonged to the *Prometheus*-family, SEA4 and SEA9 belonged to the *Enterprise*-family, SEA2 belonged to the *Galactica*-family, and SEA5 belonged to the *Phoenix*-family. Notably, SEA10 showed no sequence similarity with elements in the *starbase*.

### Prediction of functions of the SEAs

To examine the expression of genes located within SEAs, we performed RNA-seq analyses using three *A*. *oryzae* strains (RIB40, TK-35, and TK-41), which collectively harbor a broad range of SEA classes (SEA1–SEA8). Although not all cargo genes located within SEAs were transcriptionally expressed, their overall expression levels were comparable to those of genes located in other chromosomal regions (Figures S5A–S5C), suggesting that SEA-associated genes are not transcriptionally silenced and may have biological functions. To predict the functions of the identified SEAs, the genes included in the elements were annotated based on the domain structures of the proteins they encode (Data S1). The composition of cargo genes differed among the SEA classes (Figure 2A), suggesting that SEAs play different functions based on their classes. DUF3723, patatin-like phospholipase (PLP), ferric reductase (FRE), and NUDIX domain-containing genes, which are often found in *Starship* elements,^23^ were frequently included in the SEAs (Figure 2A). In a previous study, the *Starship* element *HEPHAESTUS* of *Paecilomyces variotii* was shown to comprise several genes that mediate metal resistance, while *MITHRIDATE* of *P*. *variotii* and *A*. *nidulans* were found to comprise genes that contribute to formaldehyde resistance.^24^^,^^27^ However, these genes were not included in the SEAs. Similar to *Starship* elements, we found the SEAs to frequently contain PLP genes and HET domain-containing genes, which are involved in heterokaryon incompatibility (Figure 2A).^42^ Thus, the SEAs may be involved in regulating heterokaryon incompatibility, which has been detected in *A*. *oryzae*.^43^ Furthermore, the SEAs were often found to contain DNA-binding proteins and protein kinases (Figure 2A), suggesting their potential involvement in signal transduction.

Although regions belonging to the same SEAs were highly homologous, unconserved regions containing various genes were found between several SEA pairs (Figure S3). To explore the potential relationship between *Starship* cargo genes and the industrial applications of *A*. *oryzae*, we focused on genes that may contribute to fermentation processes. Interestingly, some of the SEA regions contained α-amylase-encoding genes that are closely related to food fermentation (Figure 2B). The α-amylase genes of *A*. *oryzae* are classified into two groups, namely *amyA* and *amyB*/*C*, based on the presence of Tc1/Mariner class putative transposable element *tnpA* (acc. no. AB072434.1) downstream of *amyB*/*C*,^44^^,^^45^ and those included in the SEA region belonged to the latter group. The positions of *amyB*/*C* genes were diverse among the strains. Particularly, two *amyB*/*C* genes were divergently oriented in SEA1 of TK-41 and SEA5 of TK-35 (Figure 2B). BLASTn analysis revealed that transposons *tnpA* and *tao1* (acc. no. AB021710.1)^46^ and *jockey*-like retrotransposon (found in *Drosophila* similar to mammalian LINEs)^47^ were positioned adjacent to the α-amylase genes (Figure 2B). The *jockey*-like retrotransposons were positioned at the end of the no synteny regions containing α-amylase genes, indicating that *amyB*/*C* were translocated into specific SEAs via *jockey*-like retrotransposons. To examine whether regions containing α-amylase genes in the SEA were shared among *A*. *oryzae* strains, short-read sequence data of TK strains^37^ were mapped to the sequences of corresponding SEAs. The *amyB*/*C* genes and flanking regions in SEA3 of TK-34, SEA4_1 of TK-31, and SEA5 of TK-35 have almost no clade-specific sequences, thus, we used SEA1 of TK-41 as an example. By mapping analysis against to SEA1 of TK-41, the entire SEA1 region of TK-41 was conserved among the strains in clade G (Figure 3A), indicating that the SEA region contributes to an increase in the copy number of the α-amylase gene in strains of the same clade. Furthermore, to investigate whether the α-amylase genes located within SEA1 are functional, the two divergently oriented α-amylase genes in SEA1 of TK-41 were deleted (ΔTK41_S1-amy; Figure S6A), and α-amylase gene expression and enzymatic activity were examined. Because the nucleotide sequences of the α-amylase genes are nearly identical, the RT-qPCR analysis reflects the total transcripts of all α-amylase genes present in TK-41. The expression level of ΔTK41_S1-amy strain was decreased compared to that of the parental strain (Figure S6B). In addition, the α-amylase activity, measured by halo formation on starch agar media, was lower in the ΔTK41_S1-amy strain than in the parental strain (Figure S6C). These results support that the α-amylase genes located within SEA1 of TK-41 are transcriptionally expressed and functional.

In addition, genes encoding glycosyl transferase 8 (GT8) were detected in SEA3 of some strains (Figure 2C). To investigate the conservation of SEA3 containing GT8-encoding genes among *A*. *oryzae* strains, mapping analysis was performed. GT8 genes were distributed among the strains in clades A, C, G, and H, the mapping pattern throughout the elements was similar within the strains of the same clade (Figure 3B). Interestingly, among the 52 strains harboring GT8 genes, 27 strains are used for *miso* production and 27 strains are used for sake production, with some strains used for both applications,^37^ suggesting a possible association between the presence of GT8 genes and strain usage in these fermentation processes. Phylogenetic analysis of the DUF3435-encoding genes in SEA3 revealed separate clades between SEA3 with and without GT8 genes, and the topology of this phylogenetic tree differed from strain phylogeny based on genome scale SNPs (Figure S7A). The GT8 gene-containing SEA3 is thought to have been transposed in *A*. *oryzae* through the following sequential events: (i) origin of GT8 gene-containing SEA3 was positioned as the common ancestor of clades C and G. Clades D, E, F, and H appeared to have lost GT8 gene-containing SEA3, while the element was retained in clades C and G (Figure S7A; blue line). Based on the mapping results in Figure 3B, all clade C strains including TK-35, which was sequenced in this study, are likely to possess SEA3; therefore, GT8 gene-containing SEA3 was vertically inherited into clade C. (ii) The GT8 gene-containing SEA3 from clade G was transferred horizontally to clade C (Figure S7A; green line), leading to the presence of two copies of GT8 gene-containing SEA3 in the corresponding clade. (iii) The additional GT8 gene-containing SEA3 in clade C, acquired from clade G, was transferred horizontally to clade A (Figure S7A; green line). These results indicated that the GT8 gene-containing SEA3 was propagated mainly via horizontal transfer among *A*. *oryzae* strains. Furthermore, RNA-seq analysis showed that GT8 genes of TK-35 and TK-41 were expressed (Figures S5B and S5C), suggesting that GT8-encoding genes in SEA3 are transcriptionally active and may retain biological functions in *A*. *oryzae*. In addition, to investigate whether expression of cargo genes in SEA3 differed between donor and recipient strains of horizontal transfer, we compared the expression profiles between the putative donor TK-41 (SEA3, clade G) and recipient TK-35 (SEA3_2, clade C). The shared cargo genes were transcriptionally expressed in both strains (Figure S5D). This observation is consistent with the hypothesis that SEAs retained transcriptionally active cargo genes upon horizontal transfer, which may have contributed to strain diversification associated with industrial use of *A*. *oryzae*.

Furthermore, genes encoding a putative cellulase belonging to the glycoside hydrolase family 12 (GH12) and a putative xylanase belonging to the glycoside hydrolase family 10 (GH10), which contribute to polysaccharide degradation, specifically existed in SEA6 of TK-49 (Figure 2D). RT-qPCR analysis detected expression of the GH10 and GH12 genes, although GH12 transcripts were detected at relatively low levels (Figure S5E), suggesting that these genes are not transcriptionally silenced and may retain biological functions. GH10- and GH12-encoding genes were distributed among strains in clades D, E, and H, and strains outside of the clades (TK-24 and TK-27) (Figure 3C). Interestingly, eight of these ten strains with GH10 and GH12 genes are used for soy sauce brewing,^37^ suggesting that GH10 and GH12 genes were retained in the strains selected for soy sauce brewing.

Among the SEAs, some cargo genes were different within the same SEA class (Figure S3), indicating that during the process of strain selection according to specific applications, the retention or loss of genes related to particular functions within the SEAs may generate intra-species variation.

### Distribution of SEAs in *Aspergillus* species

To investigate the distribution of SEAs among *Aspergillus* species, their presence was examined by homology analysis of the genes included in the SEAs. SEAs were found in *A*. *flavus*, which is phylogenetically close to *A*. *oryzae*,^48^ and *Aspergillus sojae*, which is used for soy sauce fermentation,^46^ but not in other *Aspergillus* species such as *Aspergillus nidulans*, *Aspergillus niger*, and *Aspergillus fumigatus* (Figure 4A). In *A*. *flavus*, SEA2, SEA4, and SEA9 were found in multiple strains, whereas SEA7 and SEA8 were found only in La3279 and CA14 strains, respectively (Figure 4A). In contrast, SEA1, SEA3, SEA5, SEA6, and SEA10 were not detected in *A*. *flavus* (Figure 4A). SEA1, SEA3, SEA5, SEA7, and SEA9 were found in *A*. *sojae*. However, no SEAs were found in *Aspergillus parasiticus*, which is phylogenetically close to *A*. *sojae*^49^ (Figure 4A). Notably, although *A*. *oryzae* is phylogenetically farther from *A*. *sojae* than *A*. *flavus*, SEA1, SEA3, and SEA5 were shared between *A*. *oryzae* and *A*. *sojae* but not *A*. *flavus* and *A*. *parasiticus* (Figure 4A). Considering that *A*. *oryzae* and *A*. *sojae* are used in food fermentation, these SEAs may contribute to this industrial process. However, because no SEAs are conserved in *Aspergillus luchuensis* (Figure 4A), which is also used for Japanese traditional distilled liquor brewing,^50^^,^^51^ the distribution of SEAs appears to differ among industrially utilized *Aspergillus* species.

To investigate the conservation of SEA regions between *A*. *oryzae* and *A*. *flavus*, a synteny analysis was performed (Figure 4B). Phylogenetic analysis revealed that the DUF3435-encoding genes in SEA2 and *Galactica* of the *A*. *flavus* AF13 strain were phylogenetically close (Figure S2B); however, unconserved regions with different gene contents (e.g., the non-ribosomal peptide synthetase (NRPS) encoding gene G4B11_000237,^22^ which is absent in *A*. *oryzae*) were found between SEA2 of TK-41 and *Galactica* of AF13 (Figure 4B). Upon mapping against *A*. *flavus* AF13, whole sequences of *Galactica* were found to be conserved among *A*. *flavus* strains; however, the NRPS-encoding gene was not detected in the *A*. *oryzae* strains (Figure 5A; yellow arrow and highlight). Short-read mapping analysis revealed that the NRP cluster containing NRPS was either partially (clades A, B, F, and G) or entirely (clade E) absent (Figure 5A). According to the phylogenetic analysis of the DUF3435-encoding genes of SEA2, the SEA2 was thought to have been transposed in *A*. *oryzae* through the following sequential events (Figure S7B): (i) origin of SEA2 was positioned as the common ancestor of *A*. *oryzae* and *A*. *flavus* (Figure S7B; blue line). (ii) NRPS was lost in *A*. *oryzae* during differentiation from *A*. *flavus* to *A*. *oryzae* (Figure S7B; green line), and (iii) SEA2 was horizontally transferred in an independent manner at least four times among *A*. *oryzae* strains.

In contrast, SEA4 of *A*. *oryzae* contains nucleotide-binding oligomerization domain (NOD)-like receptor (NLR)-encoding genes, which are not present in SEA4 of *A*. *flavus* (Figure 4C). These NLR-encoding genes possess a structure comprising a nucleoside phosphorylase domain (IPR000845) at the N terminus, an NB-ARC domain (IPR002182) at the central region, and tetratricopeptide repeats (TPRs) (IPR019734) at the C terminus. Phylogenetic analysis of the DUF3435 #1 gene of SEA4 revealed that the SEA4 was thought to have been transposed in *A*. *oryzae* through the following sequential events (Figure S7C): (i) origin of SEA4 was positioned as the common ancestor of *A*. *oryzae* and *A*. *flavus*, and SEA4 was vertically inherited into *A*. *oryzae* strains from *A*. *flavus* (Figure S7C; blue and green lines); (ii) SEA4 without NLR genes was horizontally transferred from *A*. *flavus* into clade D; (iii) during differentiation from *A*. *flavus*, *A*. *oryzae* acquired NLR genes; and (iv) the NLR gene-containing SEA4 appears to have been horizontally transferred independently at least three times among clades in *A*. *oryzae*; however, the directionality of transfer remains unresolved (Figure S7C). The NLR genes were accompanied by a third DUF3435-encoding gene in SEA4 (DUF3435 #3), and no NLR genes were present in the regions lacking DUF3435 #3 (Figure S3; SEA4), raising the possibility that they were co-transferred with DUF3435 #3 into SEA4. Upon mapping against *A*. *oryzae* TK-35, these genes were found to be conserved in *A*. *oryzae* strains; however, *A*. *flavus* strains did not possess NLR genes (Figure 5B).

In addition to the NLR genes in SEA4, a methyltransferase (IPR029063) encoding gene was also found to be *A*. *oryzae*-specific in SEA9 (Figure 4C). Mapping against *A*. *oryzae* TK-31 revealed that the methyltransferase gene was conserved in *A*. *oryzae* strains belonging to clade B; however, *A*. *flavus* strains did not possess the methyltransferase gene (Figure 5C). Phylogenetic analysis of the DUF3435-encoding genes in SEA9 revealed that the SEA9 is inferred to have been transposed through the following sequential events (Figure S7D): (i) origin of SEA9 was positioned as the common ancestor of *A*. *oryzae* and *A*. *flavus* (Figure S7D; blue line), and (ii) SEA9 was retained only in clade B of *A*. *oryzae* and lost in the other clades of *A*. *oryzae* (Figure S7D; green line), and the methyltransferase gene was acquired at once during the differentiation from *A*. *flavus* (Figure S7D).

These findings suggest that SEAs originally present in *A*. *flavus* are mainly vertically inherited to *A*. *oryzae* and then transferred horizontally among *A*. *oryzae* strains, and that the SEAs have been evolved via gene gains/losses to have species-specific roles.

### Potential horizontal transfer of SEAs beyond genera

Investigation of SEA distributions in other filamentous fungal species also revealed the presence of SEA6 in *Monascus purpureus* KUPM5 (Figure 4A). Synteny analysis further revealed that SEA6 of *A*. *oryzae* strains exhibited high sequence similarity with the corresponding regions of chromosomes 3 and 4 in *M*. *purpureus* (Figure 6A). When whole transcript sequences were compared between *A*. *oryzae* and *M*. *purpureus*, the transcripts from SEA6 showed much higher similarity than other genes (Figure 6B), suggesting that SEA6 was transferred between these species via horizontal transfer. In addition, GH10 and GH12 genes were found in the putative SEA6 region of *M*. *purpureus* (Figure 6A). These genes were also found in SEA6 of TK-49 strain (Figure 2D). Phylogenetic analysis based on the nucleotide sequences of the DUF3435-encoding genes in *A*. *oryzae* and *M*. *purpureus* supported the following sequential evolutionary model (Figure 6C). (i) First, ancestral *A*. *oryzae* strains of clades C, D, and E acquired SEA6 containing GH10 and GH12 genes. (ii) Based on the phylogeny of DUF3435-encoding genes, SEA6 was transferred horizontally to clade H strains from the ancestor of clade D strains. (iii) SEA6 containing GH10 and GH12 genes was also horizontally transferred from the ancestors of clade D strains to *M*. *purpureus*. (iv) After the divergence of clades C and D, GH10 and GH12 genes were lost in clade C. (v and vi) From clade C, SEA6 without GH10 and GH12 genes were transferred horizontally to clade A/B and *M*. *purpureus*, respectively. Therefore, the origin of SEA6 containing GH10 and GH12 genes was inherited by multiple transfers among *A*. *oryzae* strains and from *A*. *oryzae* to *M*. *purpureus*. These results indicate that, following the loss of GH10 and GH12 genes in a part of *A*. *oryzae* strains, SEA6 without GH10 and GH12 genes were transferred horizontally among *A*. *oryzae* strains and across genera from *A*. *oryzae* to *M*. *purpureus*.

## Discussion

Transposons can alter the fitness of organisms by modifying the genome structure in response to their specific environments.^52^^,^^53^ In bacteria, the relationship between transposons and adaptive fitness has been extensively studied. ICEs are transposons that carry multiple genes within their regions and contribute to evolutionary adaptations, such as the acquisition of antibiotic resistance.^16^^,^^19^ In fungi, a functionally analogous class of elements called *Starships* has recently been identified. In this study, we used the domesticated filamentous fungus *A*. *oryzae* as a model and revealed that structural genome changes mediated by *Starships* are associated with domestication and intra-species strain variations related to their industrial applications.

First, we generated chromosome-level genome sequences (8–13 contigs) of six representative *A*. *oryzae* strains from different clades. Comparative analysis of genomic structures among *A*. *oryzae* strains in phylogenetically different clades revealed that the genome structure was largely conserved, consistent with previous studies.^54^^,^^55^ Remarkably, synteny analysis revealed the presence of *Starship* elements in *A*. *oryzae* (SEAs) ranging from 10 to 290 kbp. In short-read-based assemblies, the detection of *Starships* is challenging because of their large size.^22^ However, by generating long-read-based assemblies, we were able to identify *Starship* elements in multiple industrial strains. SEAs were classified into 10 classes based on the phylogeny of DUF3435-encoding genes and repertoires of cargo genes. Despite belonging to the same class, the chromosomal positions varied among the strains, suggesting that SEAs spread among *A*. *oryzae* strains primarily through horizontal transfer and translocation within the genome. Few studies have extensively examined the chromosomal positions of *Starships* within the same species. Gluck-Thaler et al. recently reported that *Starships* promote strain heterogeneity in the fungal pathogen *A*. *fumigatus* and observed both conserved and variable chromosomal positions of *Starship* elements.^30^ Furthermore, *Starship* element distributions are not always correlated with strain phylogeny in *A*. *fumigatus*. In *A*. *fumigatus*, a sexual life cycle has been observed,^56^ and crosses between different strains in the environment may have led to phylogeny-independent distributions of *Starship* elements. In contrast, although mosaic genome structures resulting from ancestral sexual reproduction have been reported in *A*. *oryzae*,^37^ no sexual cycle has been identified in this species to date.^57^ There is a brewing technique known as *tomokoji* (or *tomodane*) (co-cultivation of multiple strains),^58^ and it is possible that such environments have facilitated the horizontal transfer of *Starship* elements between strains. In addition, Urquhart et al. demonstrated that a *Starship* element could transpose between different fungal species by co-culture.^59^ Thus, in *A*. *oryzae*, artificial selection during intra-species strain variation for fermentation purposes may have led to differences in *Starship* presence/absence and chromosomal positions among strains of the same species.

To investigate whether SEAs were specific to *A*. *oryzae* or conserved in other *Aspergillus* species, we performed a BLASTn search. SEAs were found to be distributed only in *A*. *oryzae*, *A*. *flavus*, and *A*. *sojae* among the analyzed *Aspergillus* species. Interestingly, *A*. *oryzae* and *A*. *sojae*, which are domesticated species used for industrial applications,^46^^,^^49^ possess more DUF3435-encoding genes than *A*. *flavus*, which is consistent with the tendency for domesticated species to harbor more *Starships*.^39^ SEA1, SEA3, and SEA5 were found only in *A*. *oryzae* and *A*. *sojae*, but not in their ancestral species *A*. *flavus* and *A*. *parasiticus* (Figure 4A), suggesting that these regions may have been horizontally transferred between *A*. *oryzae* and *A*. *sojae* during domestication. SEA2, SEA4, SEA7, SEA8, and SEA9 were found in *A*. *flavus* (Figure 4A); thus, these SEAs were likely vertically inherited from the ancestral species *A*. *flavus* to *A*. *oryzae*. For instance, in SEA2/*Galactica*, the sequence identity between *A*. *flavus* strains exceeded 99%, suggesting high conservation. In contrast, in *A*. *oryzae*, recombination events and partial deletions of genomic regions or genes were observed within the same SEA class, implying that the diversification of SEAs occurred during the differentiation and domestication processes into *A*. *oryzae*. In SEA1, which is absent in *A*. *flavus* but uniquely present in *A*. *oryzae*, the region contains *AogprK2*/*AO090166000020* (Figure S3; SEA1), encoding a class VI G protein-coupled receptor, and it is known that *AogprK2* negatively regulates sclerotia formation.^60^ In *A*. *oryzae*, the majority of strains have no ability to form sclerotia,^61^ which is thought to have arisen as a result of domestication,^57^ and *AogprK2* located within SEA1 may be one of the contributing factors. Thus, cargo genes commonly carried by *Starships* that are absent in *A*. *flavus* but present in *A*. *oryzae* may underlie traits specific to *A*. *oryzae*.

SEAs contained diverse cargo genes that varied among SEA classes and strains (Figure S3; Data S1). RNA-seq analysis of three *A*. *oryzae* strains (RIB40, TK-35, and TK-41), which collectively harbor a broad range of SEA classes (SEA1–SEA8), showed that cargo genes within SEAs were not transcriptionally silenced compared with genes in other chromosomal regions (Figures S5A–S5C). This suggests that some of the cargo genes in SEAs are maintained in a transcriptionally active state and may retain biological functions.

A comparative analysis showed that both *A*. *flavus*-specific and *A*. *oryzae*-specific cargo genes were present in the SEAs. For example, while the final product remains unidentified, an NRP (non-ribosomal peptide) biosynthetic gene cluster present in SEA2 of *A*. *flavus* is partially/entirely absent in *A*. *oryzae* strains (Figures 4B and 5A). Phylogenetic analysis of the DUF3435-encoding gene suggested that the NRPS gene was lost in *A*. *oryzae* during differentiation from *A*. *flavus* or domestication (Figure S7B). Because *A*. *oryzae* strains used in fermentation were selected for the loss of aflatoxin production,^37^ it is plausible that a similar selective process contributed to the elimination of toxin-related genes during the differentiation or domestication.

In contrast, the *A*. *oryzae*-specific NLR gene was found in SEA4, and methyltransferase gene was found in SEA9 (Figure 4C). NLR genes are associated with programmed cell death, such as heterokaryon incompatibility in fungi.^62^^,^^63^ Heterokaryon incompatibility is thought to play a role in limiting the reception of harmful genetic elements such as transposons and mycoviruses between different individuals via cell fusion.^64^^,^^65^ In *A*. *oryzae*, heterokaryon incompatibility has been detected.^43^ In SEA4, the NLR gene is absent in *A*. *flavus*; however, it is present in multiple strains of *A*. *oryzae* except for clade D (Figures 5B, S3, and S7C). These NLR genes may enhance allorecognition, which contributes to genomic stability and leads to constant brewing. However, the absence of SEA4-associated NLR gene in clade D strains suggests other factors may also contribute to heterokaryon incompatibility. Additionally, DUF3435 #3 was always located adjacent to the NLR gene (Figure S3), suggesting the coexistence of additional *Starship* elements within SEA4. This suggests that *Starship* elements serve as vehicles for additional transposable elements and promote adaptation. Methyltransferases typically affect the transcription of numerous genes (such as those related to secondary metabolism, sexual/asexual development, and virulence) in *Aspergillus* sp.^66^^,^^67^^,^^68^^,^^69^ The methyltransferase gene found exclusively in *A*. *oryzae* within SEA9 may represent a unique genetic feature of clade B strains (Figure 5C). Although clade B strains are used for sake brewing, sake-brewing strains are also found in other clades. Functional characterization of methyltransferase-encoding gene in SEA9 may help clarify clade B-specific traits. These findings suggest that the presence/absence of SEAs and *A*. *oryzae*-specific cargo genes reflects selective pressures that favored strains with enhanced fermentation and metabolite production capacities during the differentiation and domestication processes.

Next, the composition of the cargo genes in the SEAs was analyzed. Synteny analysis of SEAs within the same class revealed that most cargo genes were conserved; however, extra genes were irregularly distributed in the SEAs of specific strains. To explore the relationship between SEA cargo genes and the industrial applications of *A*. *oryzae*, we focused on α-amylase, GT8, GH10, and GH12 genes because of their predicted fermentation-related functions and strain-specific distributions. For example, the α-amylase genes were located in distinct *Starship* elements such as SEA1, SEA3, SEA4, and SEA5 depending on the strains (Figure 2B). In these α-amylase containing regions, *jockey*-like retrotransposons were consistently found at the termini, possibly mediating the transfer of α-amylase gene-containing regions into SEAs (Figure 2B). In bacteria, transposons carrying genes that enhance pathogenicity or confer antibiotic resistance are inserted into ICEs, which are thought to facilitate adaptation to new niches.^70^^,^^71^ Similar to bacterial ICEs, in SEAs, the acquisition of α-amylase genes through transposons may have contributed to the adaptation of *A*. *oryzae* to the brewing industry. *A*. *oryzae* possesses multiple copies of the α-amylase encoding gene, and the copy number of them varied by clade (Figure 2B).^37^ Specifically, TK-41 in clade G have four α-amylase genes, with two copies of the α-amylase gene positioned at SEA1. Copy number of α-amylase genes in *A*. *oryzae* is almost clade-dependent as other clade G strains are similar to TK-41.^37^ Deletion of the two divergently oriented α-amylase genes within SEA1 of TK-41 reduced both α-amylase gene expression and enzymatic activity (Figure S6), demonstrating that cargo genes carried by SEAs are functional. Together with their strain-specific distribution, these findings support the possibility that functional cargo genes within SEAs may have contributed to the traits relevant to the domestication and strain variation of *A*. *oryzae*. In addition to TK-41, in other strains where the α-amylase gene is located within an SEA, the copy number of the α-amylase gene was also conserved within each clade,^37^ supporting that SEAs contribute to increase in the copy number of the α-amylase genes. TK-49, which is used for soy sauce brewing, has two copies of α-amylase genes, and they are absent from the *Starship* elements. In soy sauce brewing, where soybean rather than starch is used as the substrate,^72^ high α-amylase activity are not necessary. Collectively, *Starship* elements may have contributed to the modulation of the α-amylase gene copy number according to industrial needs. In addition to the α-amylase gene, other strain-specific genes were found within the same class of SEAs. In SEA3, GT8 genes were specifically present in clades A, C, G, and H (Figures 2C and 3B). It is noteworthy that clades A, C, and G include *A*. *oryzae* strains used for *miso* production, where high salt concentrations are encountered during fermentation.^73^ BLASTn analysis of the GT8-encoding sequences revealed similarity to genes encoding galactinol synthase (EC 2.4.1.123). The galactinol synthase enzyme synthesizes galactinol from *myo*-inositol and UDP-galactose.^74^ In plants, galactinol is known to be involved in tolerance to oxidative, heat, and salt stresses.^75^^,^^76^^,^^77^ Although the function of GT8 genes in fungi remains largely unclear, these genes may be associated with broader adaptation to fermentation-associated environmental stresses, including salt stress. In addition, GT8 genes in SEA3 of TK-35 and TK-41 were transcriptionally expressed, as shown by RNA-seq analysis (Figures S5B and S5C), suggesting that they are not transcriptionally silenced and may retain biological functions. In SEA6, the TK-49 strain contained genes encoding GH10 xylanase and GH12 cellulase (Figure 2D). Mapping to the TK-49 genome revealed that the GH10 and GH12 genes were specifically present in clades D, E, and H (Figure 3C). Many strains belonging to these clades are used for soy sauce brewing,^37^ suggesting a possible association between the presence of GH10/GH12 genes and strain application. Xylanases and cellulases are generally involved in the degradation of plant cell wall polysaccharides, including components of soybeans used in soy sauce production.^78^^,^^79^^,^^80^^,^^81^ Therefore, GH10 and GH12 genes represent candidate factors that may contribute to fermentation-related traits. RT-qPCR analysis detected expression of both GH10 and GH12 genes, although GH12 transcripts were detected at relatively low levels (Figure S5E), suggesting that these genes are not transcriptionally silenced and may retain biological functions. It is possible that these genes were preferentially maintained in strains used for soy sauce brewing; however, this hypothesis requires experimental validation. These findings suggest that SEAs would have rearranged their genetic components by acquiring genes favored through artificial selection, resulting in intra-species variation for multiple brewing applications. Consistent with this possibility, RNA-seq analyses revealed that cargo genes within SEA regions were transcriptionally expressed and were not generally silenced relative to genes in other chromosomal regions (Figures S5A–S5C). Furthermore, genes shared between the putative donor strain TK-41 and recipient strain TK-35 within the GT8-containing SEA3 were expressed in both strains (Figure S5D), suggesting that SEA cargo genes can continue to be transcriptionally expressed following horizontal transfer. Interestingly, sequences that were highly homologous to SEA6 were identified on chromosomes 3 and 4 of *M*. *purpureus* (Figure 6A). *M*. *purpureus* is traditionally used in Asia for the fermentation of foods such as *tofuyo* (Japanese fermented tofu).^82^^,^^83^ GH10 and GH12 genes, which were identified in the SEA6 region of *A*. *oryzae* TK-49, were also found on chromosome 4 of *M*. *purpureus*. Phylogenetic comparison between the DUF3435-encoding genes within SEA6 and the single-nucleotide polymorphism-based phylogeny of *A*. *oryzae* strains suggested that SEA6 originated near the roots of clades C, D, and E, underwent horizontal transfer to *M*. *purpureus*, subsequently lost the GH10 and GH12 genes, and was then transferred horizontally to *M*. *purpureus* (Figure 6C). Under laboratory conditions, hyphal fusion between auxotrophic strains of *A*. *oryzae* and *Monascus* species has been demonstrated.^84^ Although *A*. *oryzae* and *M*. *purpureus* are rarely used together in the same fermentation process, both are commonly employed in the production of fermented foods in east Asia. This geographical and ecological overlap raises the possibility that SEA6 may have been transferred horizontally between the two species via contamination or other environmental interactions. These findings, which are in line with reports on other *Starship* elements, provide evidence that SEAs are capable of horizontal transmission across genus boundaries.

We propose a conceptual model summarizing the roles of *Starships* in the differentiation and domestication to *A*. *oryzae* (Figure 7). Five SEA classes were detected in *A*. *flavus* lineages, one ancestral lineage of which differentiated into *A*. *oryzae*. During differentiation from an ancestral *A*. *flavus* lineage to *A*. *oryzae* and subsequent domestication, five additional SEA classes were acquired, whereas some SEAs appear to have been lost in individual strains or clades. In addition, some SEA cargo genes, including methyltransferase and NLR genes, were acquired, whereas SEA-associated NRP genes potentially involved in mycotoxin production were lost, generating *A*. *oryzae*-specific genetic diversification. Notably, fermentation-associated SEA cargo genes, including α-amylase, GT8, and GH10/12 genes, were also acquired, have facilitated strain variation during selection for specific industrial applications. Furthermore, SEAs have undergone intragenomic transposition and horizontal transfer among *A*. *oryzae* strains, and SEA6 appears to have been horizontally transferred to *M*. *purpureus*, a species belonging to a different genus. Together, these findings suggest that *Starship* elements serve as vehicles for the acquisition, loss, and rearrangement of genetic components, thereby generating genomic diversity during the differentiation and domestication of *A*. *oryzae* and facilitating strain variation. In yeast, an organism domesticated for multiple purposes, such as bread, sake, and wine, similar to *A*. *oryzae*, comprehensive genomic and phenotypic analyses have reported that copy number variants and gene content alterations are associated with the driving forces of domestication.^85^ *A*. *oryzae* may have adapted to an environmental niche, domestication, by employing *Starship* elements as vehicles for genetic components through the acquisition and rearrangement of cargo genes, thereby promoting strain variation for multipurpose applications. Elucidating the functions of unannotated-cargo genes within SEAs will provide further evidence for this scenario. In this study, we demonstrated that the diversity of *Starship* elements and their cargo genes between *A*. *oryzae* and *A*. *flavus*, as well as among *A*. *oryzae* strains may have contributed to the differentiation and domestication of *A*. *oryzae*. Our findings propose a eukaryotic model that diversity of genetic components within large mobile genetic elements drives the adaptation to specific environmental niches.

### Limitations of the study

Our genomic comparative analysis revealed the presence/absence of *Starship* elements among *A*. *oryzae* strains; however, because only the genome sequences of representative strains from each clade were compared, intra-clade variation could not be adequately assessed. In addition, although we identified ten classes of *Starship* elements in this study, genome sequences for some clades (e.g., clades D and H) have not yet been sequenced at chromosomal level, raising the possibility that additional uncharacterized classes of *Starship* elements remain undiscovered. Furthermore, while we found the evidence of horizontal transfer of *Starship* elements between phylogenetic clades, there are limitations in determining the directionality of transfer between donor and recipient strains. Clarifying the directionality of *Starship* transfer will require integrated phylogenetic and population-level analyses, together with expanded long-read genome sequencing of *A*. *oryzae* to facilitate more comprehensive comparative investigations.

## Resource availability

### Lead contact

Requests for further information and resources should be directed to and will be fulfilled by the lead contact, Jun-ichi Maruyama (amarujun@g.ecc.u-tokyo.ac.jp).

### Materials availability

Materials including fungal strains and plasmids generated in this study will be provided from the lead contact.

### Data and code availability

• Raw reads, genome assembly and annotation data of *A*. *oryzae* strains are deposited in the DDBJ as accession number BioProject PRJDB37648 (key resources table). The RNA-seq data of *A*. *oryzae* strains is available as the DDBJ BioProject PRJDB37768 and PRJDB42568 (key resources table).
• This study does not report original code.
• Any additional information required to reanalyze the data reported in this study is available from the lead contact upon request.

## Acknowledgments

This study was supported by the 10.13039/501100001691Japan Society for the Promotion of Science (JSPS) KAKENHI (grant numbers 18H02123 to J.M., 24H00496 to J.M., 23H04547 to J.M. and 25KJ1154 to Y.H.), JST SPRING (grant number JPMJSP2108 to Y.H.), 10.13039/501100008666Sapporo Bioscience Foundation (to J.M.) and the Sasakawa Scientific Research Grant (to Y.H.). This work was supported by the NIBB Collaborative Research Program (18-468, 19-439, 20-405 and 21-315) to J.M. We would like to thank H. Asao, A. Akita, and M. Matsumoto for next-generation sequencing at NIBB. We would like to thank Editage (www.editage.com) for English language editing.

## Author contributions

Y.H., T.K., S.S., and J.M. conceived of and designed the study. Y.H., T.K., Y.C., K.N., and K.Y. performed experiments. Y.H., K.T., Y.C. K.Y., S.S., and J.M. performed bioinformatic analysis. Y.H., T.K., K.Y., S.S., and J.M. wrote the manuscript. All authors approved the manuscript.

## Declaration of interests

The authors declare no competing interests.

## STAR★Methods

### Key resources table

**Table:** 

REAGENT or RESOURCE	SOURCE	IDENTIFIER
**Antibodies**		

*Escherichia coli* DH5α	Thermo Fisher Scientific	Cat# EC0112

**Chemicals, peptides, and recombinant proteins**		

Proteinase K Solution	KANTO CHEMICAL CO., INC	Cat# 34060-96
RNase A from Bovine Pancreas	NACALAI TESQUE, INC.	Cat# 30141-14
Pyrithiamine hydrobromide	Sigma-Aldrich	Cas# 534-64-5

**Critical commercial assays**		

QIAGEN Genomic-tips	QIAGEN	Cat# 10223
Short Read Eliminator Kit XL	Pacific Biosciences	Cat# SS-100-111-01
Oxford Nanopore Ligation Sequencing Kit	Oxford Nanopore Technologies	Cat# SQK-LSK109
Isogen	NIPPON GENE	Cat# 311-02501
RNeasy mini kit	QIAGEN	Cat# 74104
Qubit™ 1X dsDNA High Sensitivity (HS) Assay Kit	Thermo Fisher Scientific	Cat# Q33231
PrimeScript™ RT reagent Kit with gDNA Eraser (Perfect Real Time)	TaKaRa	Cat# RR047A
TB Green™ Premix Ex Taq™ II (Tli RNaseH Plus)	TaKaRa	Cat# RR820S
PrimeSTAR® HS DNA Polymerase	TaKaRa	Cat# R010A
In-Fusion® Snap Assembly Master Mix	TaKaRa	Cat# 638947
KOD FX *Neo*	TOYOBO	Cat# KFX-201
*Sma*I	TaKaRa	Cat# 1085A
*Bst*1107I	TaKaRa	Cat# 1028A

**Deposited data**		

Long read sequence data for RIB40 and TK strains	This study	DDBJ: PRJDB37648
Short read sequence data of TK strains	Watarai et al.^37^	DDBJ: PRJDB7763
Short read sequence data for RNA-seq analysis of *A. oryzae* RIB40 strain in liquid medium	This study	DDBJ: PRJDB37768
Short read sequence data for RNA-seq analysis of *A. oryzae* strains in PD agar medium	This study	DDBJ: PRJDB42568

**Experimental models: Organisms/strains**		

*A. oryzae*: Strain RIB40	Machida et al.^40^	NBRC 100959
*A. oryzae*: Strain TK-31	Watarai et al.^37^	N/A
*A. oryzae*: Strain TK-34	Watarai et al.^37^	N/A
*A. oryzae*: Strain TK-35	Watarai et al.^37^	N/A
*A. oryzae*: Strain TK-41	Watarai et al.^37^	N/A
*A. oryzae*: Strain TK-49	Watarai et al.^37^	N/A
*A. oryzae*: ΔTK41_S1-amy strain	This study	N/A

**Oligonucleotides**		

The list of oligonucleotides used in this study is shown in Table S3	N/A	N/A

**Recombinant DNA**		

pRGEgTK41_TE1amy2ND	This study	N/A

**Software and algorithms**		

MinKNOW v23.04.3	Oxford Nanopore Technologies	N/A
Tapestry v1.0.1	Davey et al.^86^	https://github.com/johnomics/tapestry?tab=readme-ov
pyGenomeViz v0.4.4	Shimoyama^87^	https://github.com/moshi4/pyGenomeViz
Fastp v0.20.1	Chen et al.^88^	RRID: SCR_016962
HISAT2 v2.2.1	Kim et al.^89^	RRID: SCR_015530
Samtools v1.14	Li et al.^90^	RRID: SCR_002105
FeatureCounts v2.0.1	Liao et al.^91^	RRID: SCR_012919
Hifiasm v0.19.5-r587	Cheng et al.^92^	RRID: SCR_021069
Funannotate v1.8.6	Palmer and Stajich^93^	RRID: SCR_023039
Porechop v0.2.4	Bonenfant et al.^94^	RRID: SCR_016967
NanoFilt v2.8.0	Coster et al.^95^	RRID: SCR_016966
Flye v2.8.0-b1695	Lin et al.^96^	RRID: SCR_017016
Pilon v1.24	Walker et al.^97^	RRID: SCR_014731
AntiSMASH fungal version 7.0	Blin et al.^98^	RRID: SCR_022060
BUSCO v5.4.4	Manni et al.^99^	RRID: SCR_015008
InterProScan v5.22-61.0	Jones et al.^100^	RRID: SCR_005829
BWA v0.7.18-r1243-dirty	Li et al.^101^	RRID: SCR_010910
GATK v4.2.0.0	McKenna et al.^102^	RRID: SCR_001876
RAxML-NG v1.2.2	Kozlov et al.^103^	RRID: SCR_022066
MUMmer v3.1	Kurtz et al.^104^	RRID: SCR_018171
Circos v0.69-6	Krzywinski et al.^105^	RRID: SCR_011798
Mafft v7.520	Katoh et al.^106^	RRID: SCR_011811
FigTree v1.4.4	University of Edinburgh; Rambaut Lab	RRID: SCR_008515
R v4.2.2	R Core Team	https://www.R-project.org/
ggplot2	Wickham	RRID: SCR_014601
chromoMap	Anand and Rodriguez et al.^107^	https://lakshay-anand.github.io/chromoMap/index.html

**Other**		

PacBio	Pacific Biosciences	N/A
MinION Mk1B	Oxford Nanopore Technologies	https://nanoporetech.com/ja/products/sequence/minion#gns.searchValue=mk1b&searchf1.value=Store&searchq.query=mk1b
Illumina Novaseq 6000 Sequencing System	Illumina	https://www.illumina.com/systems/sequencing-platforms/novaseq.html; RRID: SCR_016387
Qubit 4 Fluorometer	Thermo Fisher Scientific	Cat# Q33226
Multi-beads Shocker	Yasui Kikai	Cat# MB455U
Thermal Cycler Dice® Real Time System IV	TaKaRa	Cat#TP1000

### Experimental model and study participant details

#### *A. oryzae* strains

*A. oryzae* RIB40,^40^ TK-31, TK-34, TK-35, TK-41, and TK-49 strains^37^ were used in this study.

### Method details

#### Genomic DNA extraction in *A. oryzae*

To extract genomic DNA, conidia were inoculated into DPY medium [20 g/L dextrin, 5 g/L yeast extract, 10 g/L polypeptone, 5 g/L KH_2_PO_4_, and 0.5 g/L MgSO_4_·7H_2_O (pH 5.5)] and incubated at 30°C for 1 day. After the 1 day incubation, they were flash frozen in liquid nitrogen. Thereafter, frozen mycelia were homogenized using a Multi-beads Shocker (Yasui Kikai, Osaka, Japan). Genomic DNA of RIB40 was extracted from the homogenized mycelia using QIAGEN Genomic-tips (QIAGEN, Venlo, Netherlands), according to the manufacturer's instructions, and then used for sequencing using the PacBio SMRT sequencing (HiFi) method (Pacific Biosciences, Menlo Park, CA, USA). For sequencing using MinION (Oxford Nanopore Technologies, Oxford, UK), genomic DNAs of TK strains were extracted as described previously with some modifications.^108^ The homogenized mycelia were suspended in the extraction buffer [0.5% SDS, 50 mM EDTA, and 0.1 mg/ml proteinase K (pH 8.0)] and incubated at 60°C for 30 min. After debris removal by centrifugation, the supernatant was treated twice with phenol/chloroform/isoamyl alcohol (25:24:1) and once with chloroform/isoamyl alcohol (24:1). The extract was mixed with 4 μL of 5 mg/ml RNase A solution and incubated at 37°C for 1 h. The genomic DNA was precipitated using ethanol, and the precipitant was picked using a glass bar, washed with 70% ethanol, and dissolved in 100 μL of TE buffer. DNA concentration was measured using a Qubit 4 fluorometer (Thermo Fisher Scientific, Waltham, MA, USA).

#### Genome sequencing

For genome sequencing of the RIB40 strain, HiFi libraries were prepared using the SMRTbell Template Prep Kit 3.0 (Pacific Biosciences), according to the manufacturer’s instructions. The libraries were sequenced using the PacBio Sequel IIe platform.

For genome sequencing of the five TK strains, short DNA fragments were removed using the Short Read Eliminator Kit XL (Pacific Biosciences). Library preparation and sequencing were performed using the Oxford Nanopore Ligation Sequencing Kit (SQK-LSK109; Oxford Nanopore Technologies), MinION flow cells (R9.4.1; Oxford Nanopore Technologies), and MinION Mk1B (Oxford Nanopore Technologies), according to the manufacturer's instructions. Base calling and quality control were performed using MinKNOW v23.04.3, with default parameters.

#### RNA sequencing

For genome annotation, the RIB40 strain was incubated in DPY liquid medium at 30°C for 1 day. Frozen mycelia were homogenized using a multi-bead shocker (Yasui Kikai, Osaka, Japan). To confirm the expression of genes located within the SEAs, the RIB40, TK-35, and TK-41 strains were cultured on PD medium overlaid with cellophane at 30°C for 2 days. The harvested mycelia were frozen and subsequently homogenized using a mortar and pestle. Total RNA was extracted using Isogen (NIPPON GENE, Tokyo, Japan) and cleaned using an RNeasy Mini Kit (QIAGEN) following the manufacturer's instructions. Library preparation and sequencing using a NovaSeq 6000 were performed commercially by Novogene (Beijing, China). For RNA-seq data analysis in the RIB40, TK-35, and TK-41 strains, the sequence reads were trimmed and filtered using fastp v0.20.1,^88^ and the filtered reads were mapped to the genome sequence of the corresponding *A. oryzae* strains using HISAT2 v2.2.1.^89^ The output SAM files were converted into BAM files and indexed using Samtools 1.14.^90^ The number of reads mapped to each gene was counted using featureCounts v2.0.1,^91^ and the transcripts per million (TPM) was calculated. Log_10_(TPM + 1) values were visualized as a heatmap.

#### Genome assembly and annotation

HiFi long reads obtained by PacBio sequencing were used to generate a *de novo* assembly of *A. oryzae* RIB40 using hifiasm v.0.19.5-r587^92^ with the default settings. Misassembled short contigs (smaller than 10-kb and repeat sequences) were excluded. Genome assemblies were annotated with the RNA sequencing data using the Funannotate pipeline v1.8.16^93^ and InterProScan v5.22-61.0^100^ with default parameters.

Long reads of five TK strains (TK-31, TK-34, TK-35, TK-41, and TK-49) obtained by Nanopore sequencing were cleaned using porechop v0.2.4 for adaptor trimming^94^ and nanofilt v2.8.0 for quality filtering.^95^ The *de novo* assemblies were generated using flye v2.8.3-b1695^96^ with a nano-raw option. The assemblies were then polished with the corresponding short-read sequences (BioProject No. PRJDB7763) using Pilon v1.24.^97^ Genome assemblies of the TK strains were also annotated using Funannotate pipeline^93^ without RNA sequencing data. Biosynthesis gene clusters (BGCs) were predicted using antiSMASH fungal version 7.0.^98^

The genome completeness of the assemblies and gene annotations were evaluated with BUSCO v.5.4.4^99^ using the fungi_odb10 dataset. Telomeric repeats were identified and visualized using the weave software of the Tapestry package v.1.0.1, with -d 30 and -t TTAGGGTCAACA parameters.^86^^,^^109^

To identify DUF3435-encoding genes, protein sequences of *A. flavus* AF13 (Acc. No. GCA_014117485.1), *A. flavus* CA14 (Acc. No. GCA_014784225.2), *A. flavus* NRRL3357 (Acc. No. GCA_009017415.1), *A. flavus* NRRL21182 (Acc. No. GCA_002443195.2), *A. niger* CBS117618 (Acc. No. GCA_009176385.1), *A. fumigatus* Af293 (Acc. No. GCA_000002655.1), *A. parasiticus* CBS117618 (Acc. No. GCA_009176385.1), *A. nidulans* FGSC A4 (Acc. No. GCA_000011425.1), and *A. luchuensis* IFO4308 (Acc. No. GCA_016861625.1) were annotated InterProScan (v. 5.22-61.0).^100^ Protein sequences of *A. flavus* La3279 (Acc. No. GCA_030515275.1), *A. sojae* NBRC4239 (Acc. No. GCA_000226655.2), and *A. sojae* SMF134 (Acc. No. ASM827498v1) are publicly unavailable; these strains were annotated with the Funannotate pipeline using genome sequences of these strains. DUF3435-encoding genes were detected in the presence of PF11917 or IPR021842.

#### Phylogenetic analysis

To analyze the phylogenetic relationship between *A. oryzae* RIB40 and TK strains, a phylogenetic tree of *A. oryzae* and *A. flavus* was constructed. The publicly available short-read genomes of 35 strains in *A. oryzae* and *A. flavus* were used in this study. Short-read genomes were adapter-trimmed and quality-filtered using fastp.^88^ Next, filtered-reads were mapped to the *A. flavus* Afla-Guard (Acc. No. ASM1289687v1)^110^ by BWA-MEM^101^ and converted from sam files into bam files using SAMtools. Using GATK v4.2.0.0^102^ “Haplotype Caller” option, SNPs to Afla-Guard were called and filtered using the parameters previously described.^55^ Phylogenetic relationship was analyzed using RAxML-NG^103^ with the GTR+G+ASC_LEWIS model and 100 bootstrap replicates.

To analyze the phylogenetic relationships of *Monascus purpureus*, nucleotide sequences of the beta-tubulin, beta-actin, and calmodulin genes were connected and aligned using MAFFT, and phylogenetic analysis was performed using RAxML-NG with default parameters.

To construct a phylogenetic tree of DUF3435-encoding genes within the same class of SEAs, nucleotide sequences of DUF3435-encoding genes in representative strains were searched against the NCBI database using BLASTn analysis. Alignment by MAFFT and phylogenetic analysis were performed using RAxML-NG with default parameters.

#### Whole genome alignment

To identify the synteny between *A. oryzae* TK-31, TK-34, TK-35, TK-41, and TK-49 genomes and the RIB40 genome, MUMer aligner v3.1^104^ was used as previously described.^55^ Briefly, Nucmer was used to align with “-c 1000” parameter. The output files were filtered using delta-filter with the “-l 10000” parameter and alignments were extracted using show-coords. The alignments were written as bundle files. Using bundle files, circular synteny maps were visualized with Circos v0.69-6.^105^ Synteny analysis of genomic comparison was aligned and visualized using the pgv-mummer v.0.4.4, software of pyGenomeViz package v0.4.4, with default parameters.^87^ Large translocated regions were visualized using R package chromoMap v4.1.1.^107^ Large translocated regions and their flanking regions (15–20 kbp) were compared and visualized using the same method as in Nucmer and Circos.

#### Identification of *Starship* elements in *A. oryzae* (SEA)

Using gene prediction data, DUF3435-encoding genes were identified in six *A. oryzae* strains (RIB40, TK-31, TK-34, TK-35, TK-41, and TK-49). For phylogenetic analysis of DUF3435-encoding genes, open reading frame (ORF) sequences of DUF3435-encoding genes in *A. oryzae*, *A. flavus* (AF13, CA14, NRRL3357, La3279 and NRRL21182), *A. sojae* (NBRC 4239 and SMF134), *A. parasiticus* (CBS 117618), *A. nidulans* (FGSC A4), *A. niger* (CBS 101883), *A. fumigatus* (Af293), *A. luchuensis* (IFO4308), and *Monascus purpureus* (KUPM5; Acc. No. GCA_025999795.1) were aligned using MAFFT v7.520^106^ and analyzed using RAxML-NG.^104^ The phylogenetic tree was visualized using FigTree software.

To determine the ends of these regions, the same alleles were compared among genomic sequences of *A. oryzae* strains. Structures of SEAs or genomic syntenies within SEAs were visualized using the pgv-simpleplot or pgv-mummer software of the pyGenomeViz package v0.4.4. The annotation of genes within the SEAs was surveyed using InterProScan.

#### Identification of TIR and DR sequences

For each SEA, genomic sequences 10 kb upstream and downstream of the ends of the SEAs were retrieved and aligned using MAFFT. Direct repeat (DR) sequences were defined as > 2 bp sequences with the same strand at the end of the SEA sequence. Terminal inverted repeat (TIR) sequences were defined as sequences > 2 bp with the opposite strand at the end of the SEA sequence.

#### Conservation of SEAs among *A. oryzae* and *A. flavus* strains

To determine the conservation of SEAs among *A. oryzae* and *A. flavus* strains, short-read sequences (BioProject No. PRJDB7763, PRJNA607981, PRJNA639008, PRJNA483051, and PRJNA992514) were mapped to representative SEA sequences. The mapping depth values for the SEA regions of representative strains were normalized by dividing them by the median depth of the chromosome on which each the SEA was located and are presented as a bar graph ranging from 0 to 2. A value of approximately one indicates the presence of the corresponding region.

#### Comparison of SEAs between *A. oryzae* and other filamentous fungal species

For searching the presence of SEAs in other filamentous fungal species, whole sequences of ten SEAs of representative strains (SEA1 of RIB40, SEA2 of RIB40, SEA3 of RIB40, SEA4_1 of RIB40, SEA5 of TK-41, SEA6 of TK-34, SEA7 of TK-34, SEA8 of TK-35, SEA9 of TK-31, and SEA10 of TK-31) were used to conduct an NCBI BLASTn search using “organism” options with “*Aspergillus oryzae* (taxid:5062)”, “*Aspergillus flavus* (taxid:5059)” or excluding “*A. oryzae* and *A. flavus*”. Genomic regions with BLASTn hits above 30% in “Query Cover” and with DUF3435-encoding genes searched above were considered to indicate the presence of SEAs.

#### Analysis of gene horizontal transfer between *A. oryzae* and *M. purpureus*

To predict the horizontal transfer of SEA6, the nucleotide sequences of whole genes in *A. oryzae* TK-49 containing SEA6 were compared with those of *M. purpureus* KUPM5 using NCBI BLASTn analysis with an E-value threshold of 1e-50. For BLASTn hits, dot plots (x-axis, gene length (bp); y-axis, identity (%)) were plotted. Genomic synteny blocks were visualized using pgv-mummer. Putative genes identified to be transferred horizontally between TK-49 and *M. purpureus* are shown as red dots.

#### Investigation of the function of genes in SEAs

To classify the genes included in the SEAs, their domain structures were predicted using InterProScan. The number of genes with the same domains was counted. Then, the number of genes with domains frequently detected (DUF3435 [IPR021842 or PF11917], DUF3723 [IPR022198 or PF12520], Patatin-like phospholipase (PLP) [IPR002641 or PF01734], Ferric_reduct [IPR013121, PF08030, or IPR013130] and NUDIX [IPR015797, PF00293, or IPR000086]), heterokaryon incompatibility-related domains (WD40 G-beta repeat [IPR015943, IPR001680, PF00400, IPR020472 or IPR019775], NACHT [PF05729, IPR007111, or IPR031359] and HET [PF06985 or IPR010730]), signaling-related domains (DNA-binding; Zinc finger [IPR007087, IPR013087, or PF13920], Zn(2)Cys(6) fungal-type [PF00172 or IPR001138], RING [IPR017907, IPR013083, IPR001841, PF13923, PF00097, or PF13445], MADS [IPR002100], Homedomain-like [IPR009057, PF00046, or IPR001356], F-box [IPR001810], Pkinase [IPR011009, PF17667, IPR000719, PF00069, or IPR008271]), and other domains (Methyltransferase [PF13489, PF13649, PF08241, PF13847, PF08242] and Arrestin [IPR024391 or PF02752], Ankyrin repeats [PF13606, PF13857, PF00023, PF12796, PF13637, or IPR002110], DnaJ [PF00226 or IPR001623], Helicase [IPR014001, PF00271 or IPR001650] and α-amylase [GH13; IPR015340, PF09260, IPR006046, or PF00128]) were visualized as heatmaps drawn using R package ggplot2.

#### DNA manipulation

*Escherichia coli* DH5α strain was used for DNA manipulation. Polymerase chain reaction (PCR) for plasmid construction was performed using PrimeSTAR® HS DNA Polymerase (TaKaRa Bio, Ohtsu, Japan). The In-Fusion HD Cloning kit (TaKaRa Bio, Ohtsu, Japan) was used for plasmid construction. Genomic PCR was performed using KOD FX Neo (TOYOBO). Primers used in this study are listed in Table S3.

#### Construction of knockout strain

The *U6* promoter and a target sequence for 5'-flanking region of α-amylase gene were amplified from pRGE-gwAup^111^ using the SmaIIF1-PU6-F2nd/gTK41_TE1_amy-PU6-R1st primers and ligated with *Sma*I-digested pRGE-gRT6,^111^ yielding a genome-editing plasmid pRGEgTK41_TE1amy1st. The *U6* promoter and a target sequence for 3'-flanking region of α-amylase gene were amplified from pRGE-gwAup using the Bst-PU6F/gTK41_TE1_amy-PU6-R2nd primers, and the guide RNA and *U6* terminator were amplified from pRGEgwAup using the guide-TU6-F/Bst-TU6R primers. The two fragments were ligated with *Bst*1107I-digested pRGEgTK41_TE1amy1st, yielding a genome-editing plasmid pRGEgTK41_TE1amy2nd.

Transformation of *A. oryzae* TK-41 strain was performed as previously described.^111^ The genome-editing plasmid pRGEgTK41_TE1amy2nd and the oligonucleotide TK41_TE1_amyDN-ssDD-F were used for transformation. Dextrin-peptone (DP) medium (20 g/L dextrin, 10 g/L polypeptone, 5 g/L KH_2_PO_4_, and 0.5 g/L MgSO_4_·7H_2_O) was used for pre-culture. Czapek-dox (CD) medium (3 g/L NaNO_3_, 2 g/L KCl, 1 g/L KH_2_PO_4_, 0.5 g/L MgSO_4_·7H_2_O, and 0.002 g/L FeSO_4_·7H_2_O, and 20 g/L sorbitol [pH 5.5]) was used to select the transformants. For selection of strain carrying the pyrithiamine-resistant *ptrA* marker plasmid, 0.1 μg/ml pyrithiamine was added. To remove the genome-editing plasmid from the transformants, dextrin was added to CD medium instead of sorbitol.

#### RT-qPCR analysis

Modified DPY medium (10 g/L starch from potato, 10 g/L polypeptone, 5 g/L yeast extract, 5 g/L KH_2_PO_4_, and 0.5 g/L MgSO_4_·7H_2_O, pH 5.5) was used for TK-41 culture. To detect expression of the GH10 gene in TK-49, a modified DPY medium containing xylan from corn core instead of starch was used. To detect expression of the GH12 gene in TK-49, modified DPY medium containing carboxymethyl cellulose instead of starch was used. Mycelia were collected from liquid culture at 30°C for 1 day and immediately frozen in liquid nitrogen. Thereafter, frozen mycelia were homogenized using a Multi-beads Shocker (Yasui Kikai, Osaka, Japan). Total RNA was extracted, and cDNA synthesis was performed using PrimeScript™ RT reagent Kit with gDNA Eraser (Perfect Real Time) (TaKaRa). RT-qPCR was performed using TB Green™ Premix Ex Taq™ II (Tli RNaseH Plus) (TaKaRa) and a Thermal Cycler Dice® Real Time System IV (TaKaRa). Primers used for RT-qPCR are listed in Table S3.

#### Measurement of α-amylase activity

To measure an α-amylase activity, conidial suspensions (5.0 ×10^4^/5 μL) were spotted onto modified starch-containing DPY agar medium and incubated in the dark at 30°C. After 3 days culture, the plates were stained with iodine, and then halo sizes were measured.

### Quantification and statistical analysis

*p* values were calculated using Student’s *t*-test. *p* values less than 0.05 were considered to indicate a significant difference. ∗ *p* < 0.05, and ∗∗∗ *p* < 0.001.

## Supplemental information

Document S1. Figures S1–S7, and Tables S1–S3

Data S1. List of genes in SEAs, related to Figure S3 and S5

Data S2. Uncropped DNA gel electrophoresis images, related to Figure S6A
