# Supplementary material for: Giant transposable elements drive genetic diversification in Aspergillus oryzae, a domesticated fungus traditionally used in food fermentation
Source: iScience. 2026 Sep 1;29(9):117405. doi: 10.1016/j.isci.2026.117405 (PMC13562431; doi:10.1016/j.isci.2026.117405)
Supplement: Supplementary file 1 [file mmc1.pdf]

iScience, Volume *29*

Supplemental information

Giant transposable elements drive genetic diversification in *Aspergillus oryzae*, a domesticated fungus traditionally used in food fermentation

Yuya Hamanaka, Takuya Katayama, Yuta Chiken, Kana Nishiguchi, Katsushi Yamaguchi, Shuji Shigenobu, and Jun-ichi Maruyama

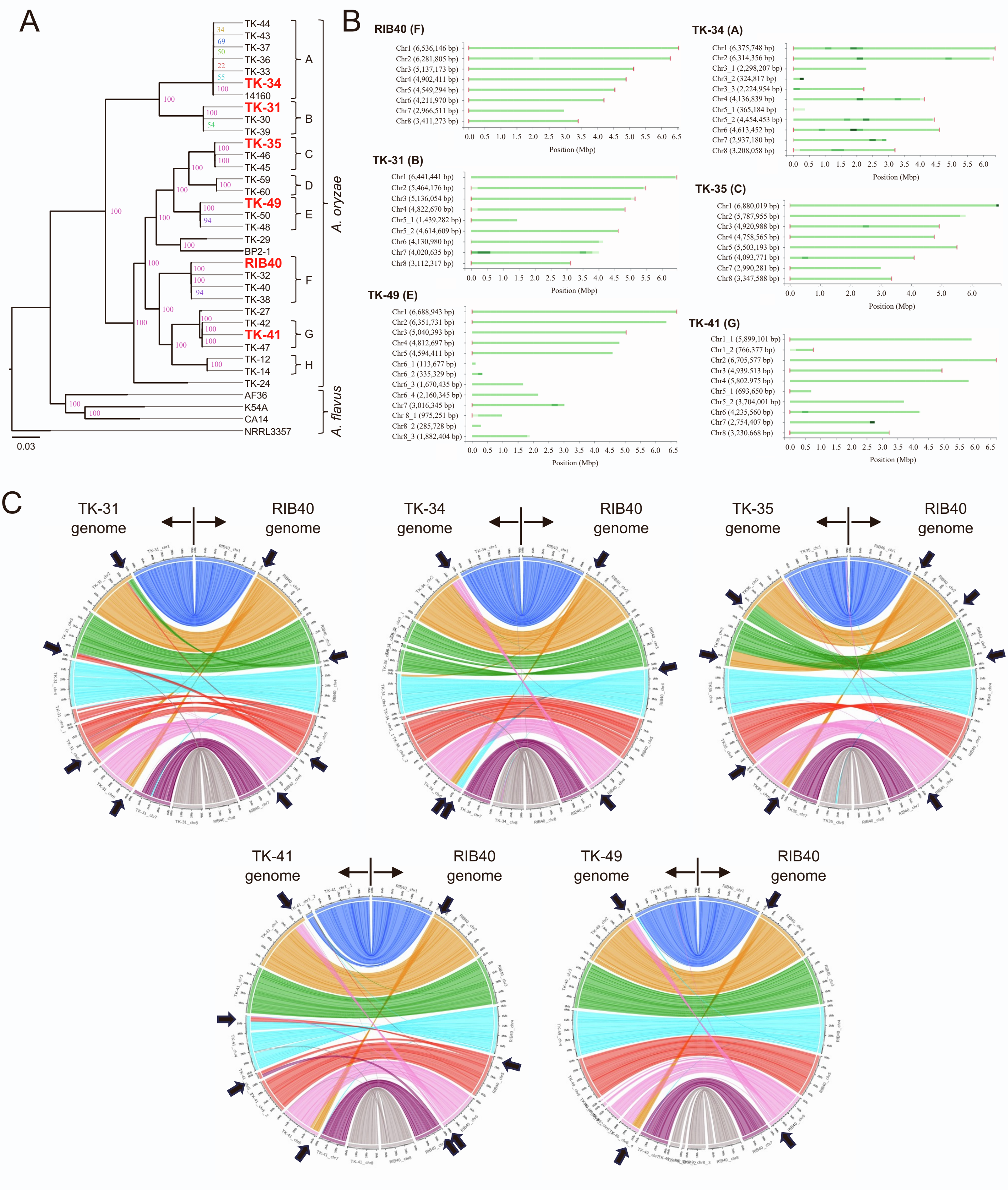

Figure S1 Genome assembly of *A. oryzae* representative strains, related to Figure 1.
(A) Phylogenetic analysis of *A. oryzae* and *A. flavus* strains. The phylogenetic tree was constructed based on the SNPs mapped against *A. flavus* Afla-Guard (ASM1289687v1)[S1]. The *A. oryzae* strains were classified into 8 clades from A to H based on Watarai et al.[S2] Numeric value in the phylogenetic tree represents bootstrap value. Strains with red color were used for genome sequencing. (B) Assemblies of six *A. oryzae* representative strains. The telomeric repeat sequence (red squares) were identified using the weave software.[S3] (C) Circular synteny map between RIB40 (right half of map) and TK strains (left half of map) genomes. Colored lines represent syntenic genomic regions (sequence length ≥ 10 kbp). Black arrows represent large translocated regions (0.3–1.0 Mbp) between two strains.

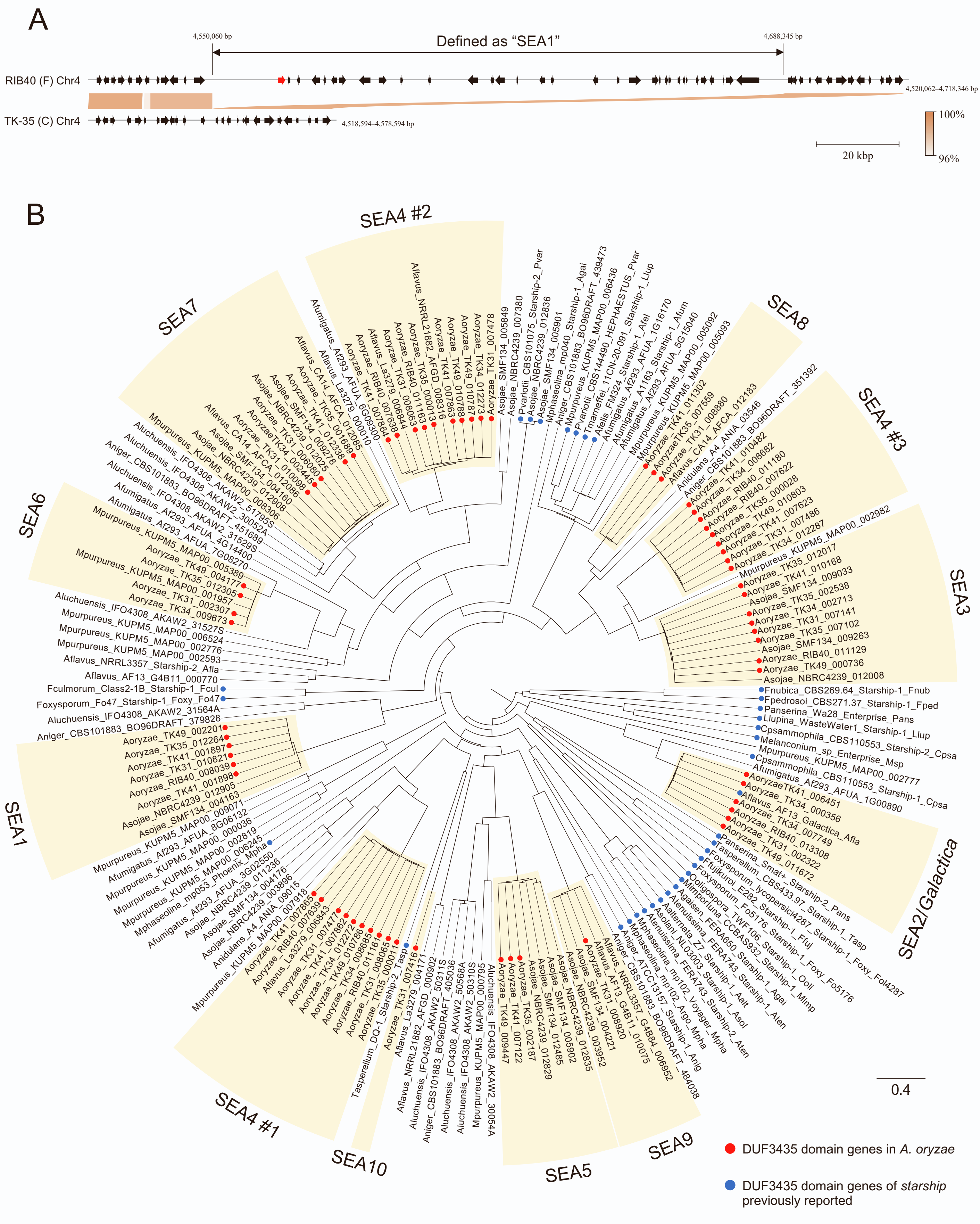

Figure S2 Identification of *Starship* elements in *A. oryzae*, related to Figure 1.
(A) Schematic diagram of definition of *Starship* element regions. Definition of SEA1 in RIB40 is shown. The sequences at chromosome 4 of RIB40 and TK-35 were compared and no synteny regions were defined as an SEA. The similarity was visualized using pgv-mummer.[S4] Red arrows represent DUF3435-encoding genes. (B) Phylogenetic tree of DUF3435-encoding genes through filamentous fungi based on the nucleotide sequences. The labels contain the names of DUF3435-encoding genes [species name_strains name_gene ID or starshipName (*cf*. Gluck-Thaler et al., 2022 Supplemental Tables S14) [S5]. Nucleotide sequences of DUF3435-encoding genes annotated by Gluck-Thaler et al.[S5] were manually retrieved from supplementary data. The clades of DUF3435-encoding genes from SEAs are highlighted.

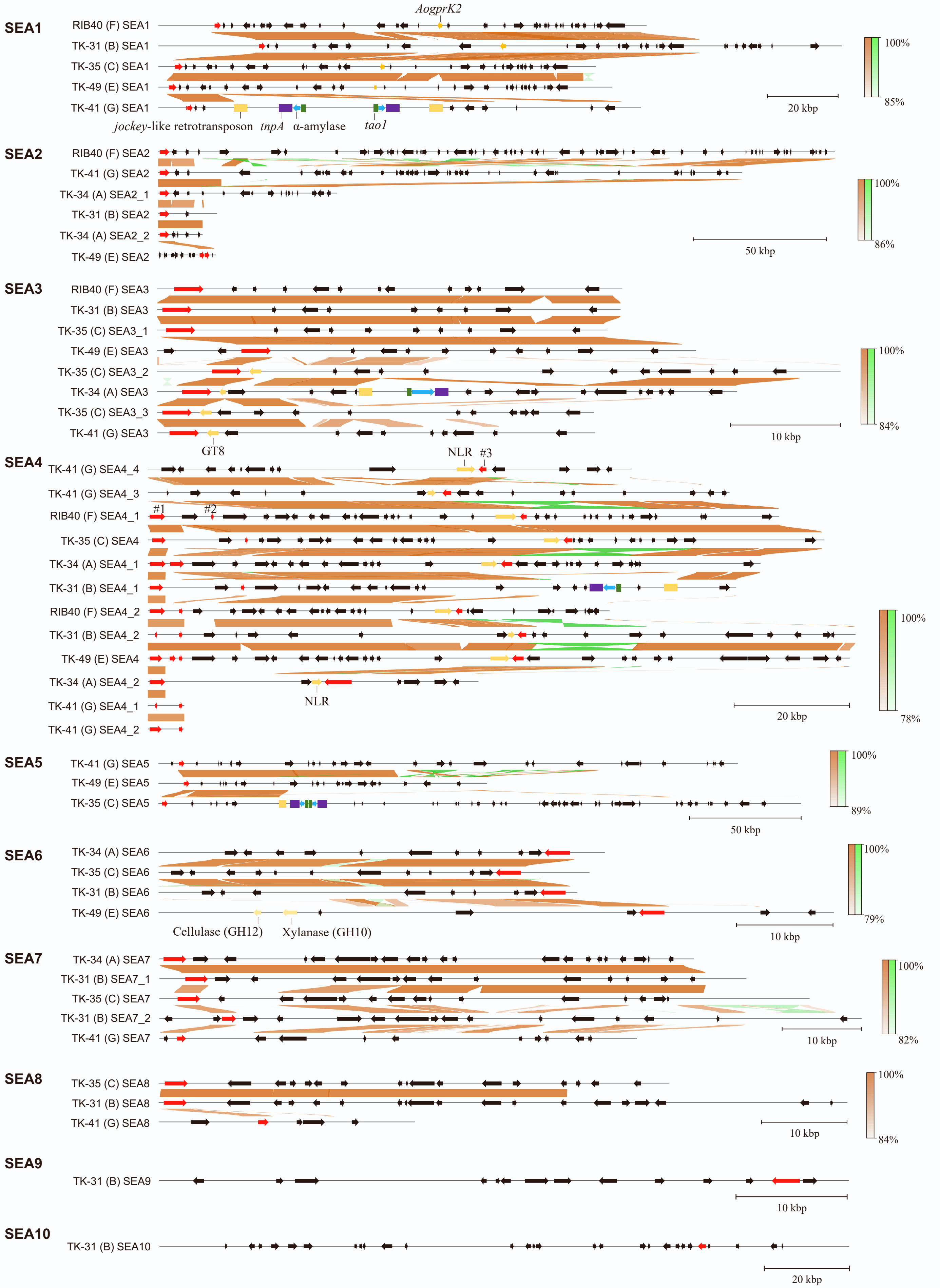

Figure S3 Synteny maps of SEAs between *A. oryzae* strains, related to Figure 1.
SEA9 and SEA10 contained only the TK-31 strain. Red arrows represent DUF3435-encoding genes. Yellow arrows represent the genes of interest. Yellow, purple, and green blocks and blue arrows indicate *jockey*-like retrotransposon, *tnpA*, *tao1*, and α-amylase genes, respectively. Similarity was visualized using pgv-mummer.[S4]

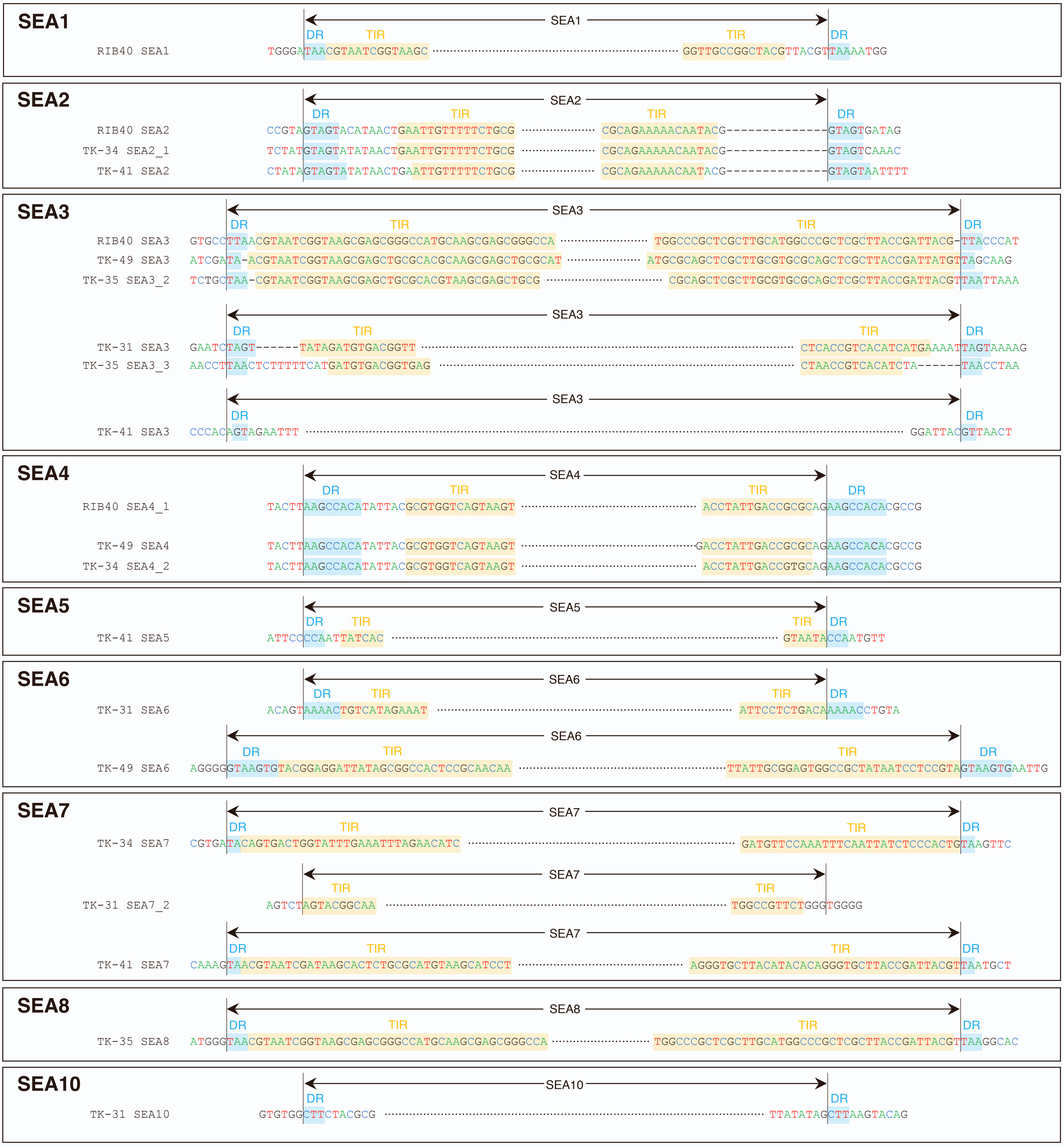

Figure S4 TIR and DR sequences in SEAs, related to Figure 1.
TIR (yellow) and DR (light blue) sequence were manually identified.

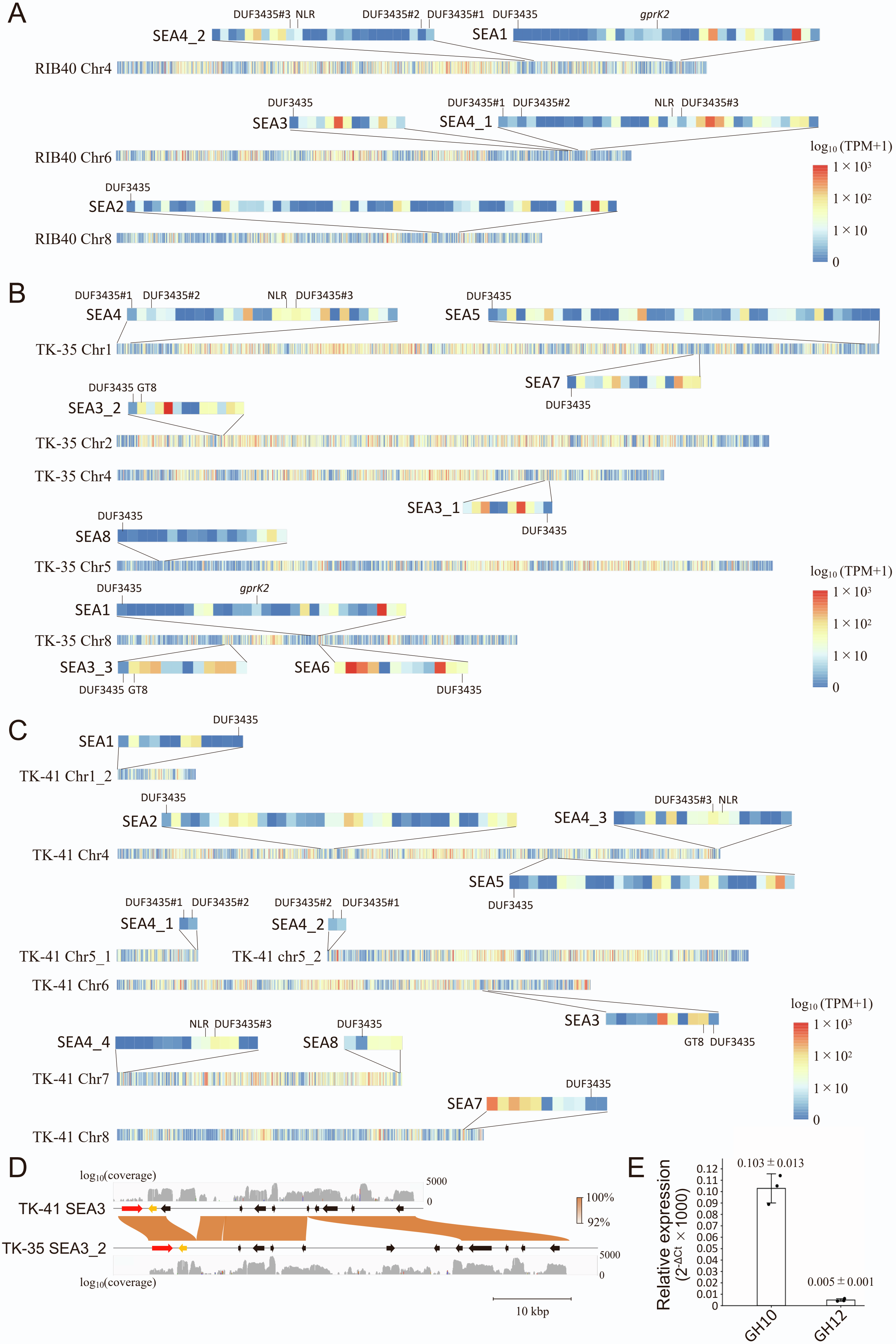

A
DUF3435#3 NLR
DUF3435#2 DUF3435#1 DUF3435
gprK2
SEA4_2
SEA1
RIB40 Chr4
DUF3435
DUF3435#1 DUF3435#2
NLR DUF3435#3
SEA3
SEA4_1
RIB40 Chr6
log10 (TPM+1)
1 × 10³
1 × 10²
1 × 10
0
DUF3435
SEA2
RIB40 Chr8
B
DUF3435#1 DUF3435#2
NLR DUF3435#3
DUF3435
SEA4
SEA5
TK-35 Chr1
SEA7
DUF3435
DUF3435 GT8
SEA3_2
TK-35 Chr2
TK-35 Chr4
SEA3_1
DUF3435
DUF3435
SEA8
TK-35 Chr5
log10 (TPM+1)
1 × 10³
1 × 10²
1 × 10
0
DUF3435
gprK2
SEA1
TK-35 Chr8
SEA3_3
SEA6
DUF3435 GT8
DUF3435
C
DUF3435
SEA1
TK-41 Chr1_2
DUF3435
SEA2
DUF3435#3 NLR
SEA4_3
TK-41 Chr4
SEA5
DUF3435
DUF3435#1 DUF3435#2
DUF3435#2 DUF3435#1
SEA4_1
SEA4_2
TK-41 Chr5_1
TK-41 chr5_2
TK-41 Chr6
SEA3
GT8 DUF3435
log10 (TPM+1)
1 × 10³
1 × 10²
1 × 10
0
NLR DUF3435#3
DUF3435
SEA4_4
SEA8
TK-41 Chr7
DUF3435
SEA7
TK-41 Chr8
D
log10(coverage)
5000
0
TK-41 SEA3
100%
92%
TK-35 SEA3_2
5000
0
log10(coverage)
10 kbp
E
Relative expression
(2-ΔCt × 1000)
0.12
0.11
0.10
0.09
0.08
0.07
0.06
0.05
0.04
0.03
0.02
0.01
0
0.103 ± 0.013
0.005 ± 0.001
GH10
GH12

Figure S5 Expression level of SEAs, related to Figures 1 and 2.

(A–C) Expression levels of genes within each SEA in RIB40 (A), TK-35 (B), and TK-41 (C). Each box of heat map represents gene expression as the $\log_{10}$(TPM+1) value. (D) The synteny map between SEA3 of TK-41 and SEA3_2 of TK-35. Red and yellow arrows represent DUF3435-encoding and GT8 genes, respectively. Coverage tracks of each SEA region, ranging from 0 to 5000 on log scale, are shown using RNA-seq data. (E) Expression levels of GH10 and GH12 genes in TK-49 (n=3). Modified DPY medium containing xylan or carboxymethyl cellulose was used to detect GH10 or GH12 gene expression, respectively. Cultures were incubated at 30˚C for 1 day. Relative expression levels were calculated as $2^{-\Delta Ct} \times 1000$ using *tef1* as the control gene. Data are shown as means ± SD.

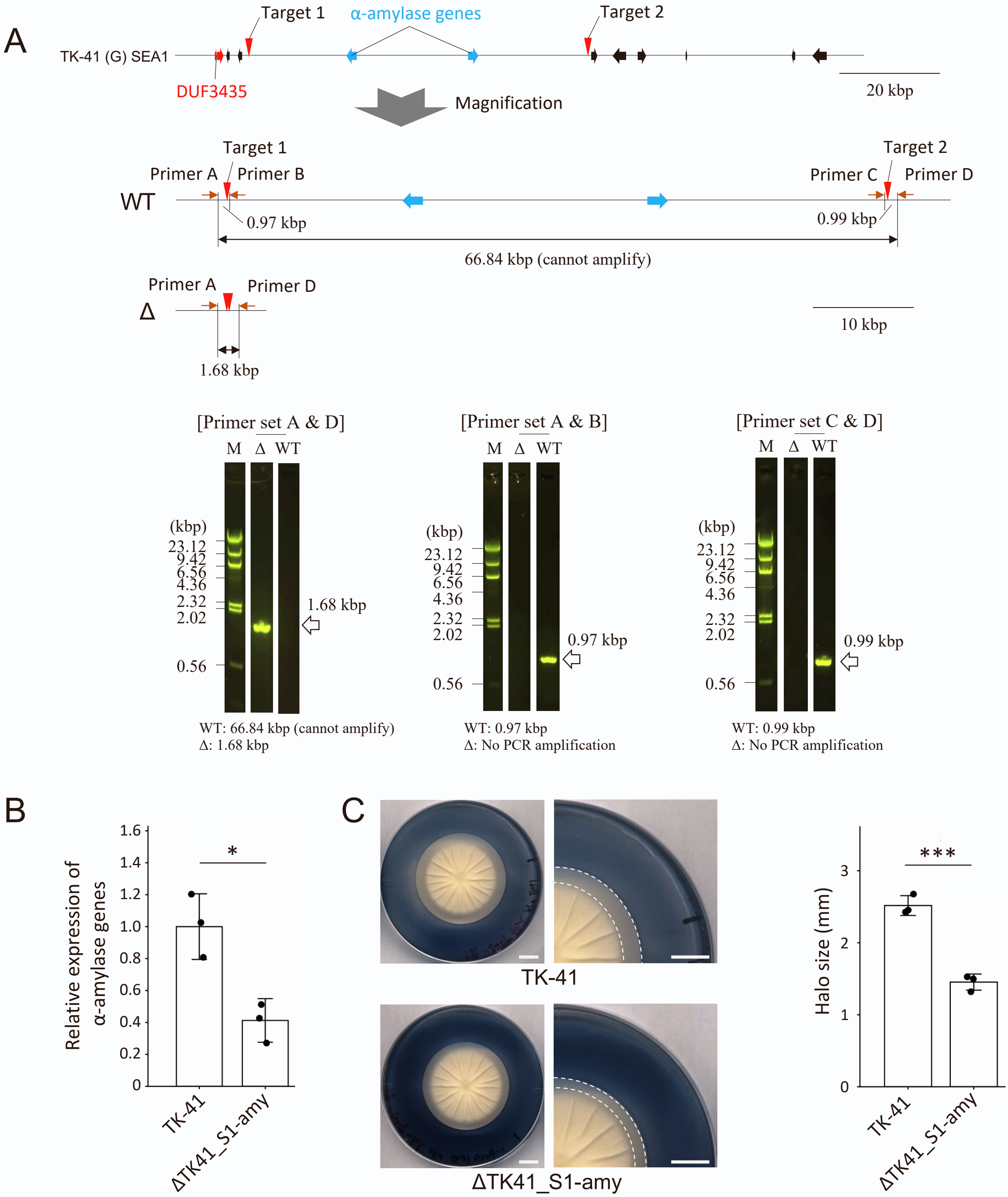

Figure S6 Functional analysis of α-amylase in SEA1 of TK-41, related to Figure 2B
(A) Construction of α-amylase gene deletion strain in SEA1. To check transformants, primers A–D (TK41_TE1_amy-F, TK41_TE1_amy-inR, TK41_TE1_amy-inF, and TK41_TE1_amy-R) were used for PCR-based verification of gene deletion. Primer pair A–D was expected to amplify a 66,840-bp fragment in the parental strain, which was too large to be amplified under the PCR conditions used, whereas a 1,684-bp fragment was amplified in the deletion strain. Primer pairs A–B and C–D amplified 971-bp and 987-bp fragments, respectively, only in the parental strain. No amplification was detected in the deletion strain using primer pairs A–B or C–D. "M" represents the λ/*Hin*dIII DNA marker. Blank spaces indicate non-adjacent lanes from the same gel. (B) Expression levels of α-amylase genes in TK-41 (n = 3). Modified starch-containing DPY medium was used for culture at 30˚C for 1 day. Relative expression levels were normalized to *tef1* expression, and the mean expression level in the TK-41 strain was set to 1. (C) Halo assay for detection of α-amylase activity after culture at 30˚C for 3 days. Images of each plate after iodine treatment are shown on the left. Scale bars represent 1 cm. White dashed lines indicate the colony and halo edges. Halo size was measured and compared between strains on the right (n = 3). *$p < 0.05$; ***$p < 0.001$; Student's *t*-test. Data are shown as means ± SD.

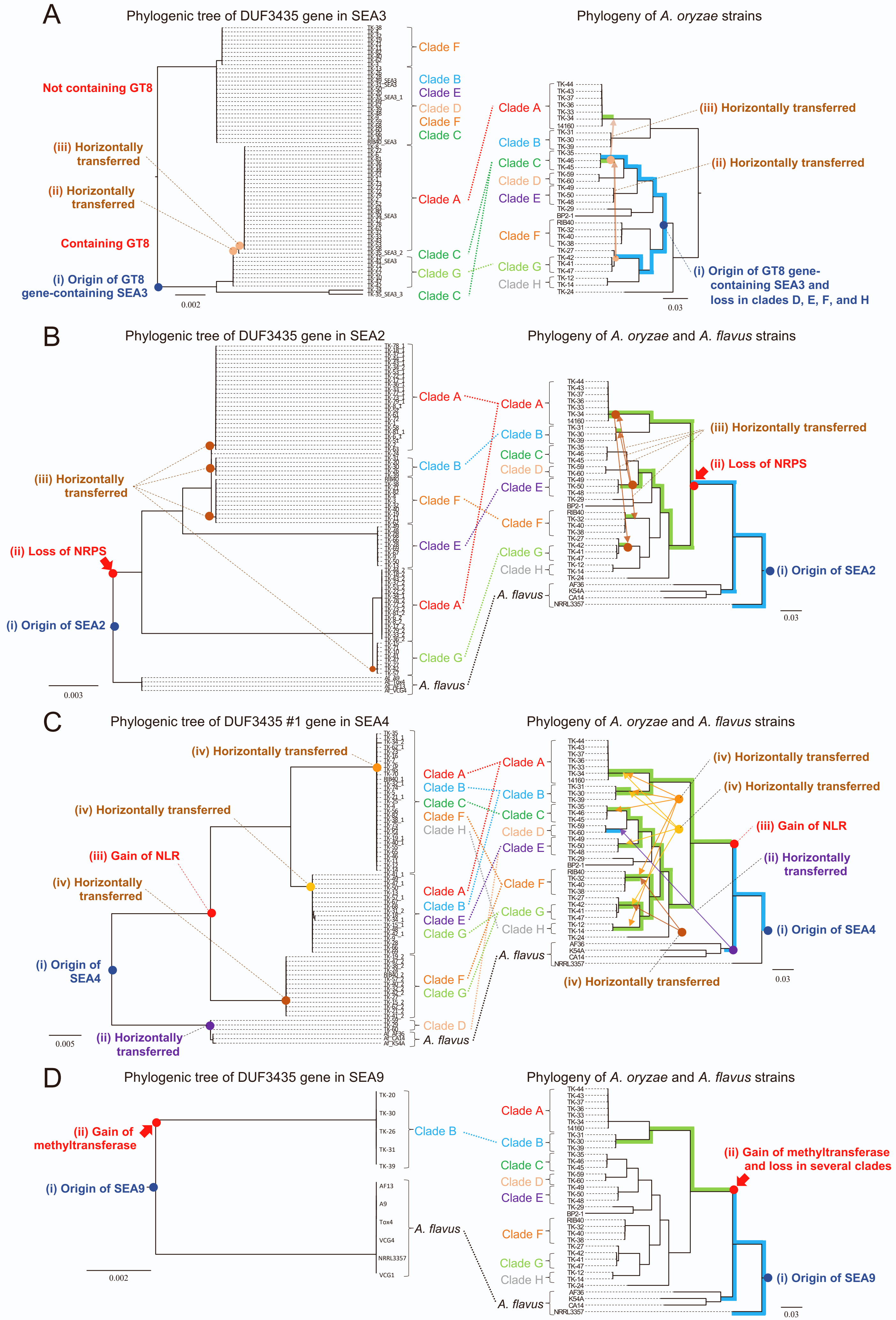

A
Phylogenic tree of DUF3435 gene in SEA3
Phylogeny of A. oryzae strains
Not containing GT8
Containing GT8
(i) Origin of GT8 gene-containing SEA3
(ii) Horizontally transferred
(iii) Horizontally transferred
Clade F
Clade B
Clade E
Clade D
Clade F
Clade C
Clade A
Clade C
Clade G
Clade C
0.002
Clade A
Clade B
Clade C
Clade D
Clade E
Clade F
Clade G
Clade H
(iii) Horizontally transferred
(ii) Horizontally transferred
(i) Origin of GT8 gene-containing SEA3 and loss in clades D, E, F, and H
0.03
B
Phylogenic tree of DUF3435 gene in SEA2
Phylogeny of A. oryzae and A. flavus strains
(iii) Horizontally transferred
(ii) Loss of NRPS
(i) Origin of SEA2
Clade A
Clade B
Clade F
Clade E
Clade A
Clade G
A. flavus
0.003
Clade A
Clade B
Clade C
Clade D
Clade E
Clade F
Clade G
Clade H
A. flavus
(iii) Horizontally transferred
(ii) Loss of NRPS
(i) Origin of SEA2
0.03
C
Phylogenic tree of DUF3435 #1 gene in SEA4
Phylogeny of A. oryzae and A. flavus strains
(iv) Horizontally transferred
(iv) Horizontally transferred
(iii) Gain of NLR
(iv) Horizontally transferred
(i) Origin of SEA4
(ii) Horizontally transferred
Clade A
Clade B
Clade C
Clade F
Clade H
Clade A
Clade B
Clade E
Clade G
Clade F
Clade G
Clade D
A. flavus
0.005
Clade A
Clade B
Clade C
Clade D
Clade E
Clade F
Clade G
Clade H
A. flavus
(iv) Horizontally transferred
(iv) Horizontally transferred
(iii) Gain of NLR
(ii) Horizontally transferred
(i) Origin of SEA4
(iv) Horizontally transferred
0.03
D
Phylogenic tree of DUF3435 gene in SEA9
Phylogeny of A. oryzae and A. flavus strains
(ii) Gain of methyltransferase
(i) Origin of SEA9
TK-20
TK-30
TK-26
TK-31
TK-39
Clade B
AF13
A9
Tox4
VCG4
NRRL3357
VCG1
A. flavus
0.002
Clade A
Clade B
Clade C
Clade D
Clade E
Clade F
Clade G
Clade H
A. flavus
(ii) Gain of methyltransferase and loss in several clades
(i) Origin of SEA9
0.03

Figure S7 Evolutionary model of transposition of SEAs, related to Figures 3 and 5.
The most plausible evolutionarily model, minimizing the number of gene gain and loss events, was estimated. Horizontal transfer was considered only between clades, excluding transfer among strains. (A) Evolutionary model of transposition of SEA3. (left) Phylogenic tree of DUF3435-encoding gene in SEA3. Following the inferred evolutionary order, the origin of GT8 gene-containing SEA3 and the horizontal transfer of GT8 gene-containing SEA3 are indicated using numbers. (right) Phylogenetic tree of *A. oryzae* strains modified from Figure S1. Note that blue lines indicate GT8 gene-containing SEA3 were retained in clades C and G from the origin of GT8 gene-containing SEA3. In addition, green lines indicate that GT8 gene-containing SEA3 from clade G was transferred horizontally to clade C and then further transferred to clade A. (B) Evolutionary model of transposition of SEA2. (left) Phylogenic tree of DUF3435-encoding gene in SEA2. Following the inferred evolutionary order, the origin of SEA2, the loss of NRPS gene, and the horizontal transfer of SEA2 are indicated using numbers. (right) Phylogenetic tree of *A. oryzae* strains (Figure S1). Blue line indicates SEA2 was retained in *A. flavus* and *A. oryzae*. During domestication from *A. flavus*, NRPS gene was lost in *A. oryzae*. Green line indicates SEA2 without NRPS gene was retained in clade A and E of *A. oryzae*, and that the elements was horizontally transferred to clade B, F and G. Note that clade A possesses additional SEA2 without NRPS gene horizontally transferred from clade E. (C) Evolutionary model of transposition of SEA4. (left) Phylogenic tree of DUF3435-encoding gene in SEA4 #1. Following the inferred evolutionary order, the origin of SEA4, the horizontal transfer of SEA4 without NLR gene, the gain of NLR gene, and the horizontal transfer of NLR gene-containing SEA4 are indicated using numbers. (right) Phylogenetic tree of *A. oryzae* strains (Figure S1). Blue and green lines indicate that SEA4 were retained in *A. flavus* and *A. oryzae*. During differentiation from *A. flavus*, NLR gene was acquired in *A. oryzae*. Note that SEA4 without NLR gene was horizontally transferred from *A. flavus* to clade D in *A. oryzae* (blue line). Green line indicates NLR gene-containing SEA4 was retained and horizontally transferred among clades in *A. oryzae* except for clade D. The thickness of green lines represents copy number of the NLR gene-containing SEA4, while the origin of these elements cannot be determined as either vertically inheritance or horizontally transfer. (D) Evolutionary model of transposition of SEA9. (left) Phylogenic tree of DUF3435-encoding gene in SEA9. Following the inferred evolutionary order, the origin of SEA9 and the gain of methyltransferase gene are indicated using numbers. (right) Phylogenetic tree of *A. oryzae* strains (Figure S1). Blue and green lines indicate that SEA9 was retained in *A. flavus* and *A. oryzae*. Note that methyltransferase gene was acquired in *A. oryzae* after differentiation from *A. flavus*. Green line indicates SEA9 containing methyltransferase gene was retained in *A. oryzae* clade B.

Table S1 Genome assembly and annotation of the *A. oryzae* strain, related to Figure S1.

	RIB40 (ASM18445v3)	RIB40 (This study)	TK-31	TK-34	TK-35	TK-41	TK-49
**PacBio and Nanopore reads**							
Number of reads	-	157,389	295,426	69,982	499,157	591,364	206,534
Total bases (bp)	-	3,090,266,464	7,281,419,324	1,593,209,926	7,028,334,993	5,410,012,933	3,180,054,713
**Genome assembly**							
Total bases in contigs (bp)	37,882,812	37,996,583	39,182,164	37,253,248	38,282,360	38,731,829	37,927,775
Number of contigs	11	8	9	11	8	10	13
Contig N50 (bp)	4,887,096	4,902,411	4,822,670	4,454,453	4,920,988	4,939,513	4,812,697
GC content (%)	47.3	47.4	47.2	47.5	47.3	47.3	47.3
BUSCOs (%) fungi_odb 10 set							
Complete and single-copy	98.3	98.2	98.3	98.2	98.4	98.4	98.0
Complete and duplicated	0.3	0.3	0.4	0.3	0.3	0.3	0.4
Fragmented	0.5	0.5	0.3	0.4	0.3	0.4	0.4
Missing	0.9	1.0	1.0	1.1	1.0	0.9	1.2
**Genome annotation**							
Protein coding genes	12,074	13,616	12,272	12,933	12,625	12,148	12,545
tRNA	242	233	237	230	250	241	243
Predicted secondary metabolite clusters	-	76	81	81	79	78	81
BUSCOs (%) fungi_odb 10 set							
Complete and single-copy	96.3	99.5	96.6	97.9	97.6	95.9	97.5
Complete and duplicated	0.3	0.3	0.3	0.4	0.3	0.3	0.4
Fragmented	0.0	0.3	1.6	1.1	1.1	2.4	0.9
Missing	3.4	0.1	1.5	0.6	1.0	1.4	1.2

Table S2 List of ten *Starship* Elements in *A. oryzae* (SEA), related to Figure S3.

SEA	Strain	SEA name	Captain gene	Chromosome	Start	End	Length (bp)	GC content (%)
SEA1	RIB40	RIB40_SEA1	RIB40_008039	RIB40_chr4	4,550,060	4,688,345	138,286	41.9
	TK-31	TK-31_SEA1	TK31_010821	TK31_chr7	1,776,914	1,970,495	193,582	40.2
	TK-35	TK-35_SEA1	TK35_012264	TK35_chr8	1,652,608	1,776,470	123,863	43.3
	TK-49	TK-49_SEA1	TK49_002201	TK49_chr1	6,508,121	6,636,712	128,592	41.9
	TK-41	TK-41_SEA1	TK41_001898	TK41_chr1_2	1	136,632	136,632	43.1
SEA2	RIB40	RIB40_SEA2	RIB40_013308	RIB40_chr8	2,536,689	2,790,445	253,757	47.4
	TK-41	TK-41_SEA2	TK41_006451	TK41_chr4	1,728,915	1,947,800	218,886	48.4
	TK-34	TK-34_SEA2_1	TK34_000356	TK34_chr1	931,015	998,122	67,108	47.0
	TK-31	TK-31_SEA2	TK31_002322	TK31_chr2	660,634	682,762	22,129	46.7
	TK-34	TK-34_SEA2_2	TK34_007749	TK34_chr5_2	1	16,656	16,656	45.1
	TK-49	TK-49_SEA2	TK49_011672	TK49_chr7	2,994,681	3,016,431	21,751	46.6
SEA3	RIB40	RIB40_SEA3	RIB40_011129	RIB40_chr6	3,747,323	3,789,658	42,336	45.9
	TK-31	TK-31_SEA3	TK31_007141	TK31_chr5_1	184,135	226,332	42,198	46.7
	TK-35	TK-35_SEA3_1	TK35_007102	TK35_chr4	3,765,579	3,806,580	41,002	46.5
	TK-49	TK-49_SEA3	TK49_000736	TK49_chr1	2,271,562	2,320,627	49,066	45.5
	TK-35	TK-35_SEA3_2	TK35_002538	TK35_chr2	875,494	937,705	62,212	43.4
	TK-34	TK-34_SEA3	TK34_002713	TK34_chr2	1,215,530	1,268,394	52,865	47.1
	TK-35	TK-35_SEA3_3	TK35_012017	TK35_chr8	870,209	910,000	39,792	46.9
	TK-41	TK-41_SEA3	TK41_010168	TK41_chr6	3,318,863	3,358,666	39,804	47.6
SEA4	RIB40	RIB40_SEA4_1	RIB40_011161	RIB40_chr6	3,844,234	3,953,624	109,391	46.8
	TK-35	TK-35_SEA4	TK35_000011	TK35_chr1	49,983	167,245	117,263	46.8
	TK-34	TK-34_SEA4_1	TK34_012272	TK34_chr8	746,539	852,729	106,191	46.8
	TK-31	TK-31_SEA4_1	TK31_008065	TK31_chr5_2	1,672,853	1,774,835	101,983	45.7
	TK-41	TK-41_SEA4_1	TK41_007862	TK41_chr5_1	687,321	693,650	6,330	46.6
	TK-41	TK-41_SEA4_2	TK41_007865	TK41_chr5_2	1	6,330	6,330	46.2
	TK-41	TK-41_SEA4_3	TK41_007623	TK41_chr4	5,702,151	5,802,975	100,825	46.8
	RIB40	RIB40_SEA4_2	RIB40_007639	RIB40_chr4	3,324,438	3,404,515	80,078	45.7
	TK-31	TK-31_SEA4_2	TK31_007477	TK31_chr5_1	1,316,618	1,439,282	122,665	46.3
	TK-49	TK-49_SEA4	TK49_010786	TK49_chr7	302,340	423,995	121,656	46.6
	TK-34	TK-34_SEA4_2	TK34_008685	TK34_chr5_2	2,599,280	2,656,586	57,307	47.7
	TK-41	TK-41_SEA4_4	TK41_010482	TK41_chr7	1	83,846	83,846	46.9
SEA5	TK-41	TK-41_SEA5	TK41_007122	TK41_chr4	4,011,088	4,270,795	259,708	46.0
	TK-49	TK-49_SEA5	TK49_009447	TK49_chr6_3	1	147,228	147,228	43.0
	TK-35	TK-35_SEA5	TK35_002187	TK35_chr1	6,591,974	6,880,019	288,046	45.4
SEA6	TK-34	TK-34_SEA6	TK34_009673	TK34_chr6	861,889	908,135	46,247	41.1
	TK-35	TK-35_SEA6	TK35_012305	TK35_chr8	1,776,523	1,821,150	44,628	41.4
	TK-31	TK-31_SEA6	TK31_002307	TK31_chr2	600,144	643,518	43,375	42.9
	TK-49	TK-49_SEA6	TK49_004177	TK49_chr2	5,829,533	5,899,479	69,947	35.9
SEA7	TK-34	TK-34_SEA7	TK34_002445	TK34_chr2	426,158	493,662	67,505	48.3
	TK-31	TK-31_SEA7_1	TK31_010098	TK31_chr6	3,356,131	3,430,282	74,152	44.9
	TK-35	TK-35_SEA7	TK35_001689	TK35_chr1	5,160,914	5,243,094	82,181	42.4
	TK-31	TK-31_SEA7_2	TK31_006080	TK31_chr4	1,568,276	1,656,993	88,718	42.2
	TK-41	TK-41_SEA7	TK41_012338	TK41_chr8	3,027,232	3,087,564	60,333	46.7
SEA8	TK-35	TK-35_SEA8	TK35_007559	TK35_chr5	311,927	371,645	59,719	45.6
	TK-31	TK-31_SEA8	TK31_008880	TK31_chr5_2	4,148,258	4,228,738	80,481	45.3
	TK-41	TK-41_SEA8	TK41_011302	TK41_chr7	2,701,165	2,731,129	29,965	48.5
SEA9	TK-31	TK-31_SEA9	TK31_008920	TK31_chr5_2	4,335,364	4,397,477	62,114	44.8
SEA10	TK-31	TK-31_SEA10	TK31_007416	TK31_chr5_1	966,649	1,129,027	162,379	34.4

Table S3 List of oligonucleotides, related to STAR Methods.

Name	Sequence 5' – 3'	Description
**Primers for construction of gene deletion**		
SmaIIF-PU6-F2nd	TCGAGCTCGGTACCCTTTTA	Amplification of the *U6* promoter and a target sequence for 5' flanking region of α-amylase gene.
gTK41_TE1_amy-PU6-R1st	TTCTAGCTCTAAAACTGACCCCGCCATGGTGACACACTTGTTCTTCTTTACAATGATTTATTT	
Bst-PU6F	AGCTTATTTTTGTATAATGCCGGCTCATTCAAAC	Amplification of the *U6* promoter and a target sequence for 3' flanking region of α-amylase gene.
gTK41_TE1_amy-PU6-R2nd	TTCTAGCTCTAAAACTAGCTTGGCGGCCGCTGAGCACTTGTTCTTCTTTACAATGATTTATTT	
guide-TU6-F	GTTTTAGAGCTAGAAATAGCAAGTTAAAATAAGGC	Amplification of the guide RNA and *U6* terminator.
Bst-TU6R	TATCACAAAACAGTAAGCAGCTCTATATCACGTGACG	
TK41_TE1_amyDN-ssDD-F	TTAAGTACGGTAGAACATTGGATACCTATCCGATGGAAAAGGTGGAAGCTAATACAAACA	For transformation of *A. oryzae* as a doner DNA.
TK41_TE1_amy-F	CGTGATGCAGAGCAGTCTTATAAAAGG	For checking the transformants of *A. oryzae*.
TK41_TE1_amy-R	GCTGTAGTAGACTCGCATTATTCTAGG	
TK41_TE1_amy-inF	GGATATTCACCACTCAGGATAGCTG	
TK41_TE1_amy-inR	GTTCGTTCGAATCCACTGATCCATTG	
**Primers for qRT-PCR**		
amyB-41-F	CAGACTCAACACTCCTGGGC	Amplification of the α-amylase genes in TK-41.
amyB-41-R	GAATGCTGCGACGTTCTTGG	
GH10-49-F	GGCAAAGTCTACGCTTGG	Amplification of the GH10 gene in TK-49.
GH10-49-R	TGGAAAAGACGCTGTCAC	
GH12-49-F	TAACTCAGGCGTGGACTTCG	Amplification of the GH12 gene in TK-49.
GH12-49-R	AATCACCGCTGGATGTAGCA	
TMtef1FW	AGCCCATGTGTGTGGAGTCTT	Amplification of *tef1*.
TMtef1RV	AACCGACTTGATAACTCCGACG	

Supplemental references

[S1] Fountain, J.C., Clevenger, J.P., Nadon, B., Wang, H., Abbas, H.K., Kemerait, R.C., Scully, B.T., Vaughn, J.N., and Guo, B. (2020). Draft genome sequences of one *Aspergillus parasiticus* isolate and nine *Aspergillus flavus* isolates with varying stress tolerance and aflatoxin production. Microbiol. Resour. Announc. *9*, e00478-20. https://doi.org/10.1128/MRA.00478-20.

[S2] Watarai, N., Yamamoto, N., Sawada, K., and Yamada, T. (2019). Evolution of *Aspergillus oryzae* before and after domestication inferred by large-scale comparative genomic analysis. DNA Res. *26*, 465–472. https://doi.org/10.1093/dnares/dsz024.

[S3] Davey, J.W., Catta-Preta, C.M.C., James, S., Forrester, S., Motta, M.C.M., Ashton, P.D., and Mottram, J.C. (2021). Chromosomal assembly of the nuclear genome of the endosymbiont-bearing trypanosomatid *Angomonas deanei*. G3 (Bethesda) *11*, 1–7. https://doi.org/10.1093/g3journal/jkaa018.

[S4] Shimoyama, Y. (2024). pyGenomeViz: A genome visualization python package for comparative genomics. https://github.com/moshi4/pyGenomeViz.

[S5] Gluck-Thaler, E., Ralston, T., Konkel, Z., Ocampos, C.G., Ganeshan, V.D., Dorrance, A.E., Niblack, T.L., Wood, C.W., Slot, J.C., Lopez-Nicora, H.D., et al. (2022). Giant *Starship* elements mobilize accessory genes in fungal genomes. Mol. Biol. Evol. *39*, msac109. https://doi.org/10.1093/molbev/msac109.
