# Supplementary material for: Giant transposable elements drive genetic diversification in Aspergillus oryzae, a domesticated fungus traditionally used in food fermentation
Source: iScience. 2026 Sep 1;29(9):117405. doi: 10.1016/j.isci.2026.117405 (PMC13562431; doi:10.1016/j.isci.2026.117405)
Supplement: Supplementary file 2 [file mmc3.pdf]

Data S2. Uncropped DNA gel electrophoresis images, related to Figure S6A.

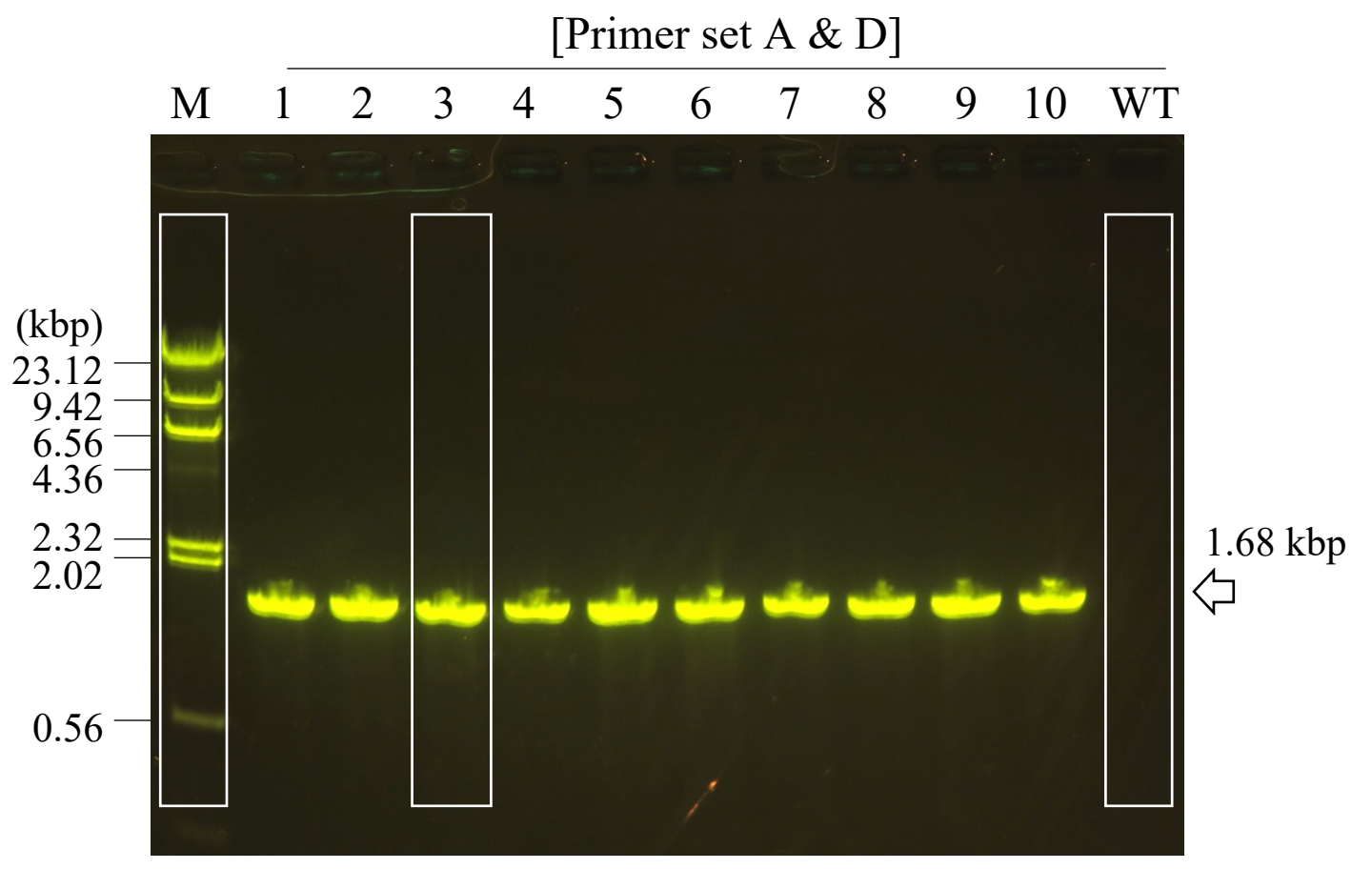

WT: 66.84 kbp (cannot amplify)
Δ: 1.68 kbp

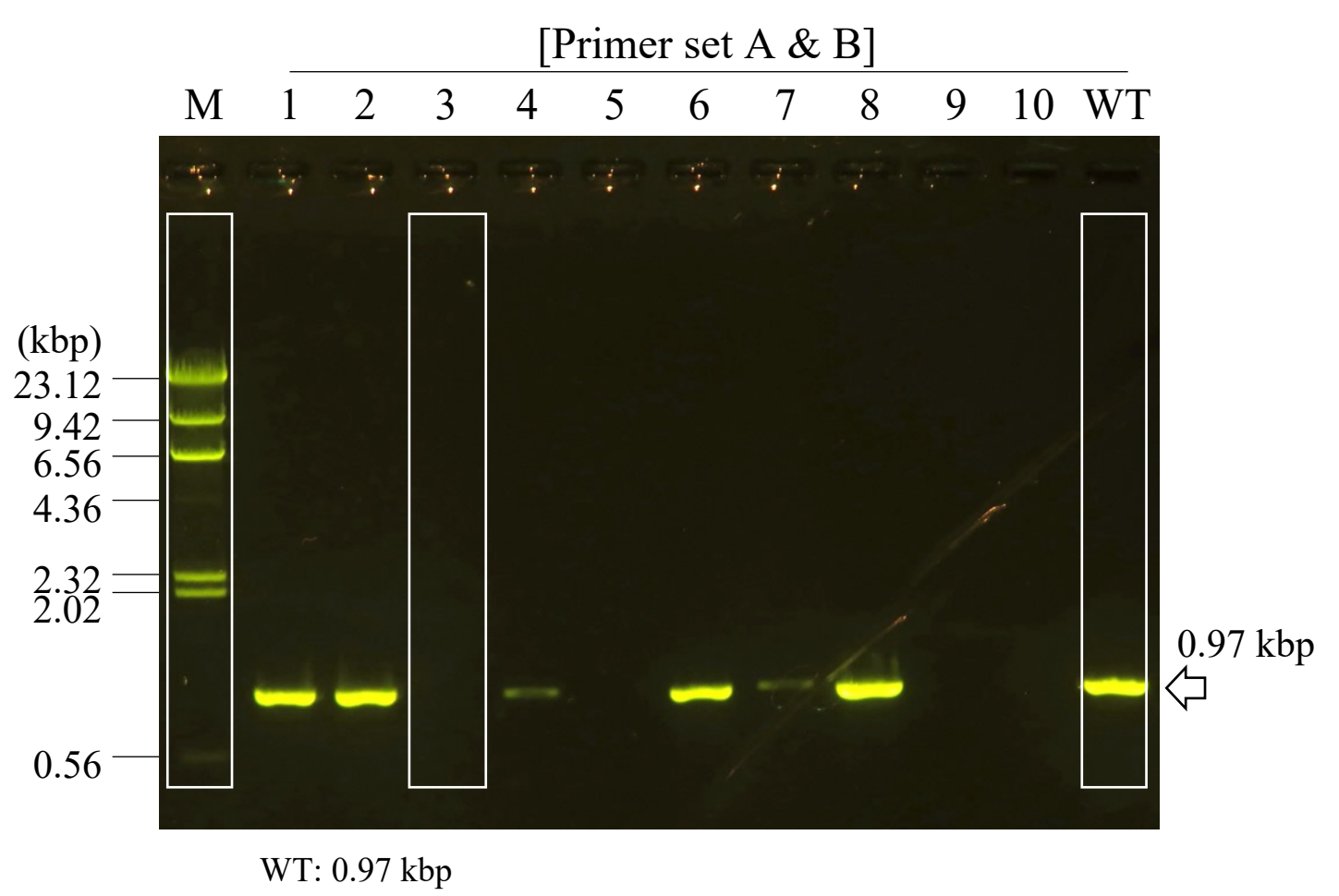

WT: 0.97 kbp
Δ: No PCR amplification

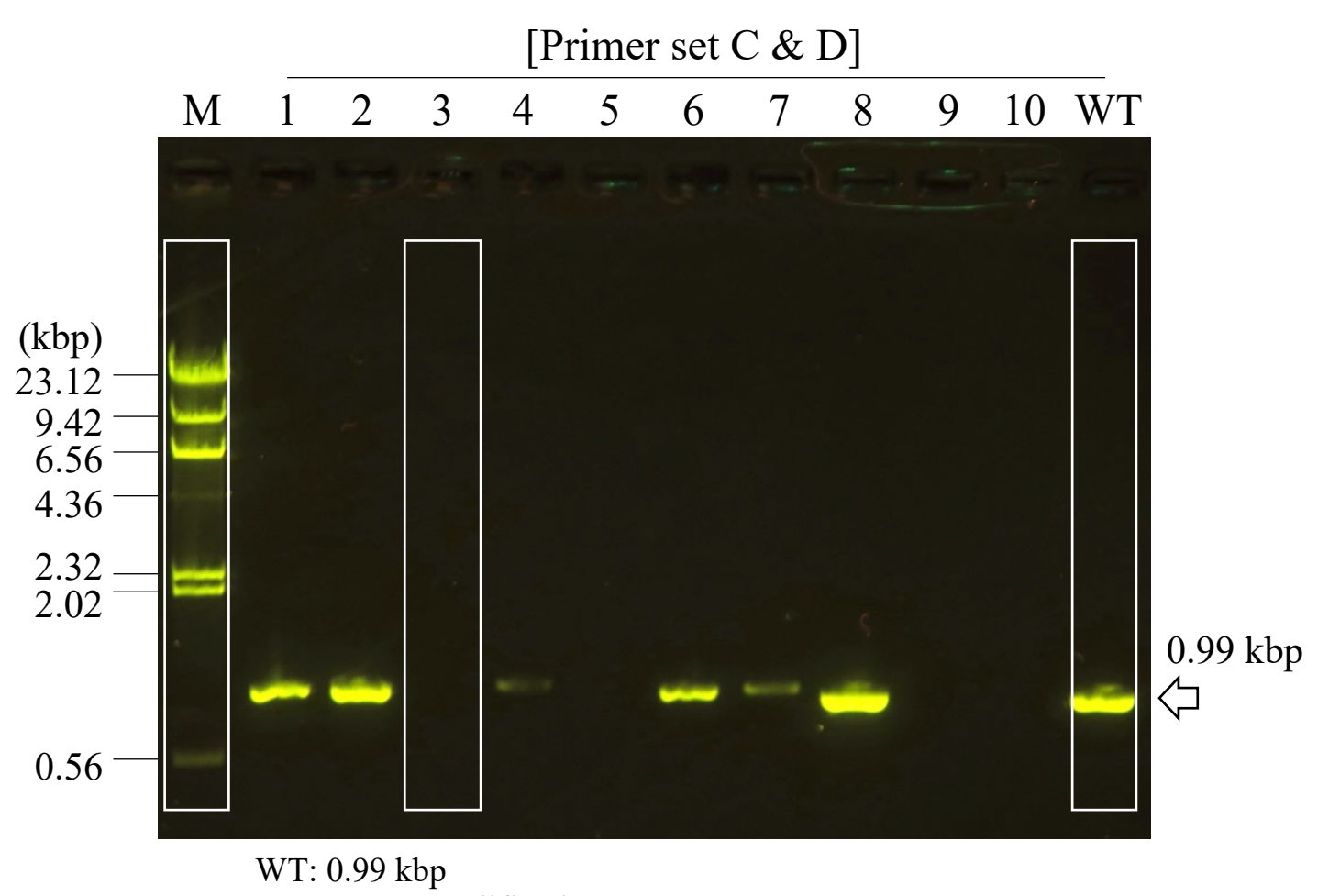

WT: 0.99 kbp
Δ: No PCR amplification
